# Not5-dependent co-translational assembly of Ada2 and Spt20 is essential for functional integrity of SAGA

**DOI:** 10.1093/nar/gkw1059

**Published:** 2016-11-29

**Authors:** Sari Kassem, Zoltan Villanyi, Martine A. Collart

**Affiliations:** Department of Microbiology and Molecular Medicine, Faculty of Medicine, Institute of Genetics and Genomics Geneva, University of Geneva, Rue Michel-Servet 1, 1211 Geneva 4, Switzerland

## Abstract

Acetylation of histones regulates gene expression in eukaryotes. In the yeast *Saccharomyces cerevisiae* it depends mainly upon the ADA and SAGA histone acetyltransferase complexes for which Gcn5 is the catalytic subunit. Previous screens have determined that global acetylation is reduced in cells lacking subunits of the Ccr4–Not complex, a global regulator of eukaryotic gene expression. In this study we have characterized the functional connection between the Ccr4–Not complex and SAGA. We show that SAGA mRNAs encoding a core set of SAGA subunits are tethered together for co-translational assembly of the encoded proteins. Ccr4–Not subunits bind SAGA mRNAs and promote the co-translational assembly of these subunits. This is needed for integrity of SAGA. In addition, we determine that a glycolytic enzyme, the glyceraldehyde-3-phosphate dehydrogenase Tdh3, a prototypical moonlighting protein, is tethered at this site of Ccr4–Not-dependent co-translational SAGA assembly and functions as a chaperone.

## INTRODUCTION

The transition of the chromatin from a compact state to an active state is mandatory for transcription. Post-translational modifications on histone tails contribute to interchange chromatin states and represent a major mechanism of eukaryotic transcription regulation. Enzymes that modify the histone tails often exist in multiprotein assemblies. The 1.8 MDa multi-subunit SAGA complex is such a transcriptional coactivator that regulates 10% of the yeast genome. It is composed of 19 subunits and bears 2 enzymatic activities: acetylation of histones H3 and H2B via the Gcn5 histone acetyltransferase (HAT) and deubiquitylation of histone H2B via the Ubp8 deubiquitinating enzyme (DUB). Besides these histone modifying functions, SAGA also binds to transcriptional activators and facilitates the assembly of the pre-initiation complex. It enhances the recruitment of RNA Polymerase II (RNAPII) via direct interaction with the TATA binding protein TBP and it shares a subunit with TREX2, an mRNA export complex (reviewed in (1–5)).

**Figure 1.**
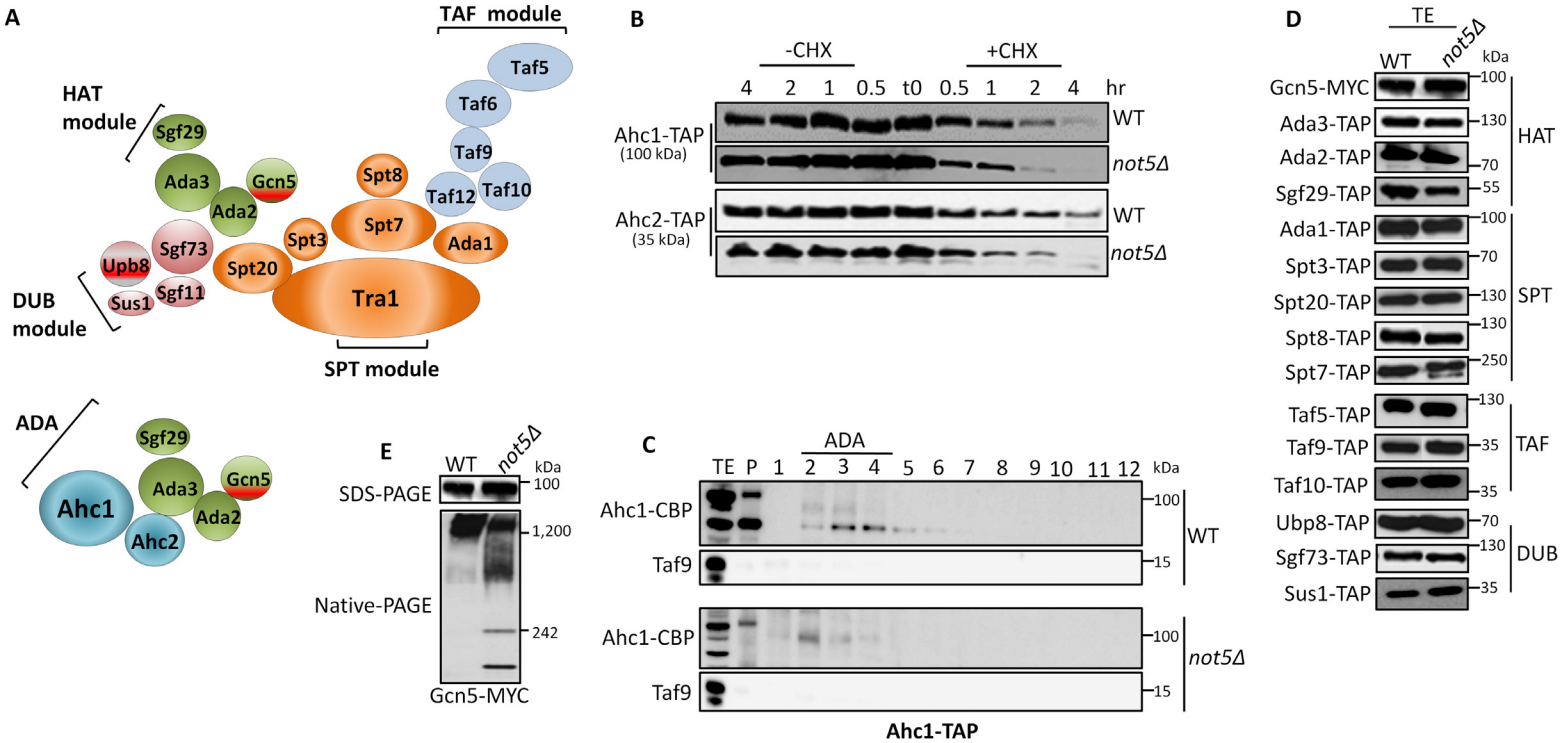
Gcn5 complexes are compromised in the absence of Not5. (**A**) Cartoon of SAGA and ADA as modular complexes according to (6). (**B**) ADA subunits are unstable in *not5Δ*. Wild-type (WT) and *not5Δ* cells expressing Ahc1-TAP or Ahc2-TAP were grown to exponential phase (time 0) and then collected at the different time points indicated (0.5, 1, 2 and 4 h) after treatment with cycloheximide (+CHX) or without addition of cycloheximide (−CHX). Total protein extracts from these aliquots were separated on sodium dodecyl sulphate-polyacrylamide gel electrophoresis (SDS-PAGE) and transferred to membranes that were revealed with PAP antibodies. (**C**) Lack of ADA in *not5Δ*. Ahc1-TAP was purified by a single affinity purification step from WT and *not5Δ* and purified proteins were separated on a 1–20% sucrose gradient. Proteins from the different fractions were TCA precipitated and loaded on SDS-PAGE, and transferred to a membrane that was probed with anti-CBP or anti Taf9 as indicated. Fraction 1 is the lightest and fraction 12 the heaviest. The position of elution of ADA is indicated. (**D**) Equal expression of SAGA subunits in *not5Δ*. 20 μg of total extracts (TE) from WT and *not5Δ* expressing SAGA subunits fused to a C-terminal Tap-tag (TAP) or MYC-tag as indicated were separated on SDS-PAGE and transferred to membranes that were revealed with PAP or MYC antibodies. Equal loading can be seen by the ponceau staining of the blots on Supplementary Figure S1B. (**E**) Gcn5 complexes are disrupted in *not5Δ*. TE from cells expressing Gcn5-MYC were separated by SDS-PAGE (upper panel) or Native-PAGE (lower panel) that were transferred to membranes for western blot analysis with MYC antibodies.

According to one study, SAGA is organized in four modules (Figure 1A) (6). A first central SPT module contains a scaffold protein Tra1, 2 TBP binding proteins, Spt3 and Spt8 and three structural subunits of the complex, Spt7, Ada1 and Spt20. A second TAF module is composed of five TBP associated factors (Tafs) that are shared with the general transcription factor TFIID. These are Taf5, Taf6, Taf9, Taf10 and Taf12. A third DUB module carries one of the two enzymes of SAGA, the Ubp8 deubiquitinase and three additional subunits, Sgf73 that anchors the module to Spt20, Sgf11 and Sus1 that is also a subunit of the TREX complex important for mRNA export. A fourth HAT module carries the second enzymatic activity, acetylation. It is made of the enzyme Gcn5, Ada2 that anchors the module to the rest of SAGA, Ada3 and Sgf29. The HAT and DUB modules can be isolated as stable sub-complexes from purifications using tagged subunits of HAT or DUB in cells lacking Spt20. The HAT module is also part of a different complex called ADA containing the Ahc1 and Ahc2 proteins (6,7).

Two recent studies have provided a somewhat different vision of SAGA assembly (8,9). The first indicated that the Taf subunits together with Ada1 and Spt7, form the central core of the complex as they do in TFIID. The second described great flexibility for SAGA and an arrangement of the complex in three layers, Tra1 above, an Spt-Taf-Spt sandwich in the middle where the DUB module is located and the HAT module in a lower layer (10).

Despite this information about the structure of SAGA by electron microscopy (11,12), the pathway of assembly of the individual subunits into the complex has not been characterized. As for most multi-protein complexes we do not know whether it is assembled post-translationally, or whether subunits meet their partners while still being translated at the ribosome. This latter type of co-translational assembly has been suggested to be wide-spread (13).

Ccr4–Not is a conserved eukaryotic complex that in the yeast *Saccharomyces cerevisiae* consists of nine subunits and bears two enzymatic functions: ubiquitination mediated by Not4, a ring E3 ligase and deadenylation mediated by Ccr4 and Caf1, the major eukaryotic deadenylase. Not1 is the scaffolding protein of the complex. The Ccr4–Not complex also harbors multiple non-enzymatic subunits (Not2, Not3, Not5, Caf40 and Caf130 in yeast) that have nevertheless been linked to several key steps of gene expression (reviewed in (14–16)). Originally the *NOT* genes were isolated in a genetic selection as transcriptional repressors that could distinguish between core promoters (17–19), and since then several studies have linked in particular the NOT module to transcription. More recently the Ccr4–Not complex was shown to bind transcription elongation complexes and promote resumption of elongation from a backtracked RNAPII (20).

The Ccr4–Not complex has been connected to SAGA in many different ways. For instance the N-terminal region of Not2 was shown to associate with Ada2 (21) and the deletion of Spt3, a subunit of the SAGA core, was found to suppress transcriptional phenotypes associated with mutations in Not1. This suppression correlated with increased recruitment of SAGA subunits to core promoters (22). SAGA controlled genes are TATA-containing and highly induced, and they were reported to be preferentially affected upon mutation of the Ccr4–Not complex (23). In particular 35% of SAGA-controlled genes were observed to be downregulated in *not5Δ*. The deletion of either Not4 or Not5, like deletion of the SAGA subunits Gcn5, Spt7, Ada2 and Spt20, were defined in a genome-wide screen to be defective in global levels of histone H3 acetylation (24). It was determined that Gcn5 purified from *not5Δ* extracts was unable to acetylate nucleosomal substrates, a function that requires Gcn5 to be incorporated in HAT complexes (25–28). Finally, a chromatin immunoprecipitation-sequencing (ChIP-seq) study showed that subunits of Ccr4–Not are recruited to the open reading frames of SAGA-regulated genes (29).

In this work we investigated at a molecular level how the Ccr4–Not complex and SAGA were functionally connected. We show that in *not5Δ* SAGA integrity is compromised. We see that full-length Spt20 purifies less with other SAGA subunits and that Sgf29 of the HAT module is less incorporated into SAGA. We determine that subunits belonging to different modules of SAGA, namely Ada2 of the HAT module and Spt20 of the core module, are assembled co-translationally. We further show that *GCN5, SPT20* and *ADA2* mRNAs are tethered together and that Not5 is important to retain Ada2 at this site of Spt20 production. In turn this allows Gcn5 to efficiently associate with Ada2 and Spt20, allow correct SAGA assembly and nuclear localization of Gcn5.

## MATERIALS AND METHODS

### Strains and plasmids

The *S. cerevisiae* strains used in this work are listed in Supplementary Table S1. Genes were deleted or modified to encode C-terminally tagged proteins using one-step polymerase chain reaction (PCR) methods described in (30,31). The full list of the oligonucleotides used in this study is presented in Supplementary Table S2. We generated plasmids expressing N-terminally tagged proteins using the drag and drop system and pGREG516 (32). This was also used to change promoters, coding sequences, and terminators. The list of plasmids used is presented in Supplementary Table S3.

### SAGA purification

Proteins were isolated by a single affinity purification step as described (33) with some modifications. Briefly, 3 liters of yeast cells expressing TAP-tagged proteins were grown at 30°C in YPD to an OD_600_ of 2.0. The washed pellet was resuspended with SAGA lysis buffer (40mM HEPES-KOH PH 8.0, 350 mM NaCl, 10% glycerol) and flash frozen with liquid nitrogen as drops. Cell drops were broken with a MM 400 CyroMill (Retsch) at 30 pulses/min for 2 min. The generated cell powder was dissolved in SAGA lysis buffer supplemented with 0.5 mM DTT, 1 mM PMSF and 2 mg/ml protease inhibitor cocktail (Roche) and spun at 6000 *g* for 10 min at 4°C. Supernatants were cleared by centrifugation in a Beckman Ti60 rotor (40 000 rpm, 30 min, 4°C). 400mg of total lysate was incubated with IgG sepharose beads (GE Healthcare). Proteins were bound by rotating at 4°C for 2 h and subsequently washed with 10 ml SAGA lysis buffer and 10 ml Tobacco Etch Virus protease (TEV) buffer (10 mM Tris–HCl, pH 8.0, 150 mM NaCl, 0.1% NP-40, 0.5 mM ethylenediaminetetraacetic acid (EDTA), 10% glycerol, 1 mM DTT). TEV protease (100 U) cleavage was performed in 1 ml of TEV buffer at 30°C for 1h.

### Polysome fractionation

Preparation of total extracts (TE) and polysome fractionation was done exactly as described previously (34).

### Co-immunoprecipitation and RIP

TE were prepared as described above. Aliquots were kept aside for western blotting. For RIP experiments, 8 mg of the TE was incubated with 50 μl of equilibrated IgG sepharose beads (GE Healthcare) in a rotator for 2 h at 4°C. The proteins bound to the beads were washed twice with 200 μl of lysis buffer and twice with 200 μl of TEV buffer. Beads were then sedimented, resuspended in 200 μl of TEV buffer and 1 unit of TEV protease (Invitrogen) and incubated 1 h at 30°C. 25% of the TEV eluate (supernatant) were kept aside for western blotting and the rest was used for RNA extraction. RNA was isolated by adding an equal volume of TriZol (Invitrogen). Samples were mixed and incubated for 20 min on ice then 0.3 volumes of chloroform were added. After centrifugation, the upper phase was collected and the RNA precipitated with 0.7 volumes of isopropanol and 3 μl of linear acrylamide (Fermentas). Pellets were resuspended in H2O and were DNaseI treated (RQ1 RNase-free DNase, Promega). A total of 300 ng of the RNA from TEV eluates and from TE (isolated separately) were reverse transcribed with M-MLV RT (Promega) using oligo d(T) primers according to the manufacturer's instructions. A total of 5 μl of the first strand cDNA solution were mixed with 10 μl ABsolute qPCR SYBR Green Mix (ABgene), 0.2 μl forward primer (50 μM) and 0.2 μl reverse primer (50 μM) in a 20 μl reaction. qPCR was performed using the PCR parameters: ((95°C, 10 min) then (94°C, 15 sec) and (60°C, 1 min) for 35 cycles). Relative enrichment ratios of mRNAs in the immunoprecipitation (IP) were determined by calculating the relative difference: ΔCt = Ct _(Input)_ – Ct _(IP)_ and then using a primer efficiency of 2: 2^ΔCt^. All qPCR primers that were used were constructed to amplify approximately 200 bp long fragments and were close to the poly (A) tail of the mRNA. The RNase inhibitor (RNasin, Fermentas) was applied at several steps of the RIP at 80 units/ml. The primers are listed in Supplementary Table S2. RIPs were calculated as enrichment over the total mRNA pool from which the RIP was performed.

### RIP from polysome fractions

Fractions 13, 14 and 15 from the sucrose gradients that correspond to heavy polysomes according to the A_254_ absorbance read by the UV/Vis detector UA6 (Teledyne Isco) were pooled. The same process of co-immunoprecipitation method described above was followed. Reverse transcription was performed. RIP with the ribosomal subunit (Rpl17-TAP) in both wild-type (WT) and *not5Δ* was used to calculate enrichment relative to translatability as it reflects the total polysome mRNA and total mRNA pool from which the RIP was performed. Relative enrichment ratios of mRNAs in the IP from polysomes were determined by calculating the relative difference: ΔCt = Ct _(IP via Rpl17-TAP)_ – Ct _(IP via SAGA-TAP)_ and a primer efficiency of 2: 2^ΔCt^. RNasin Plus (Promega) at 0.2 unit/μl was added at several steps of the RIP.

### Two-hybrid experiments

Plasmids expressing the C-terminus of Not1 fused to the Gal4 DNA binding domain, Tdh3 fused to the Gal4 activation domain and their cognate empty plasmids (pOBD) and (pAD2) derivatives in different combinations were introduced by transformation into the yeast strain YSH625 described in (35). Cells containing the relevant combination of plasmids were grown to the same optical density (OD_600_ of 1.0) and serial 5-fold dilutions were spotted onto agar plates selective for plasmids and the reporter gene. Plates were incubated for 4 days at 30°C.

### Immunofluorescence

A total of 5 ml of exponentially growing yeast were fixed with 600 μl of 37% formaldehyde at RT for 1 h. The cells were collected and the pellet was washed twice with phosphate buffered saline containing 0.1% tween 20 and resuspended in 1 ml of spheroplasting buffer (20 mM potassium phosphate pH 7.4, 1.2 M sorbitol). 0.2 ml of cell suspension was treated with 2 μl of 1.42 M β-mercaptoethanol and 20 μl of 1 mg/ml lyticase (Sigma) for 10 min at 30°C. A total of 20 μl of the spheroplasts suspension were immobilized on polylysine coated microscope slides. Immunostaining was performed using a rabbit polyclonal antibody against CBP (Sigma) at a 1:50 dilution as primary antibody or a rabbit polyclonal antibody antibody against Yra1 (kind gift from F. Stutz) at 1:100 and Alexa Fluor 488 anti-Rabbit IgG (Life technologies) at a 1:1000 dilution as secondary antibody and DAPI was used at a 1:2000 to mark the nucleus. Images were taken using a Confocal Laser Scanning Microscopy (Leica SP5). Images were obtained by optical sectioning (z-stacks) with a step size of 0.2 μm and further processed with ImageJ-win64.

### Native gel analysis

A total of 0.1 mg of total protein obtained from 100 OD units of cells growing exponentially were loaded on Native polyacrylamide gel electrophoresis (PAGE) 3–12% Bis-Tris gels (Invitrogen) and were analyzed by western blotting.

### Antibodies

The following antibodies were used: Peroxidase-Anti-Peroxidase (P1291; Sigma) to detect the protein A domain of TAP, anti-CBP against the Calmodulin Binding Protein domain of TAP (DAM1411288; Millipore), a monoclonal anti-HA antibody (H3663; Sigma), a monoclonal anti-c-MYC antibody (M5546; Sigma). Polyclonal antibodies against Taf9, Spt3 and Not5 were produced in rabbits (Elevage Scientifique des Dombes, France) and used at a 1:5000 dilution. The secondary antibodies were anti-Mouse-HRP (IgG-Peroxidase conjugate; A9044; Sigma) used at 1: 10 000 or anti-Rabbit-HRP (IgG-Peroxidase conjugate; A8275; Sigma) used at 1:10 000.

## RESULTS

### The integrity of Gcn5 complexes is compromised in cells lacking Not5

To understand why Gcn5 nucleosomal HAT activity that depends upon Gcn5 incorporation into ADA or SAGA complexes is defective in *not5Δ* (24,36,37), we first explored the impact of Not5 on expression of subunits specific to the ADA complex, namely Ahc1 and Ahc2 (6,7). We noted that the proteins were equally expressed in WT and mutant cells, but that they were less stable in cells lacking Not5 (Figure 1B). Affinity purification followed by separation of purified proteins on a sucrose gradient revealed that the Ahc1 purified from WT cells was mainly present in fractions 2–5 of the gradient with a peak in fractions three and four (Figure 1C) whereas it was only detected in fraction 2 in *not5Δ*. This difference in the mutant is compatible with a lack of ADA complexes in *not5Δ* that probably explains also the reduced stability of Ahc1 and Ahc2 shown above (Figure 1B).

We then explored the expression of SAGA subunits. All SAGA subunits tested were equally expressed in WT and *not5Δ* (Figure 1D). Moreover stability assays indicated that the deletion of Not5 didn't alter the half-life of the major components of SAGA (Supplementary Figure S1). Since unaltered expression of SAGA subunits could not explain defective SAGA HAT activity in *not5Δ*, we next investigated the integrity of the SAGA complex. Extracts from WT or *not5Δ* cells expressing separately each of the SAGA subunits with a tag were analyzed by native PAGE. Most SAGA subunits were detected only at the top of the native gel with an apparent size in the MDa range compatible with SAGA (see examples in Supplementary Figure S2). In mutant cells, this was mostly also the case, but we noted that there was generally less signal for these large complexes on the native gels when we compared the mutant to the WT, and this despite equal expression of the proteins visible on sodium dodecyl sulphate-polyacrylamide gel electrophoresis (SDS-PAGE) (Supplementary Figure S2). This was also the case for Gcn5 (Figure 1E). In addition Gcn5 from *not5Δ* was more spread out at the top of the native gel and it was additionally present in a smaller complex of approximately 250 kDa (Figure 1E).

**Figure 2.**
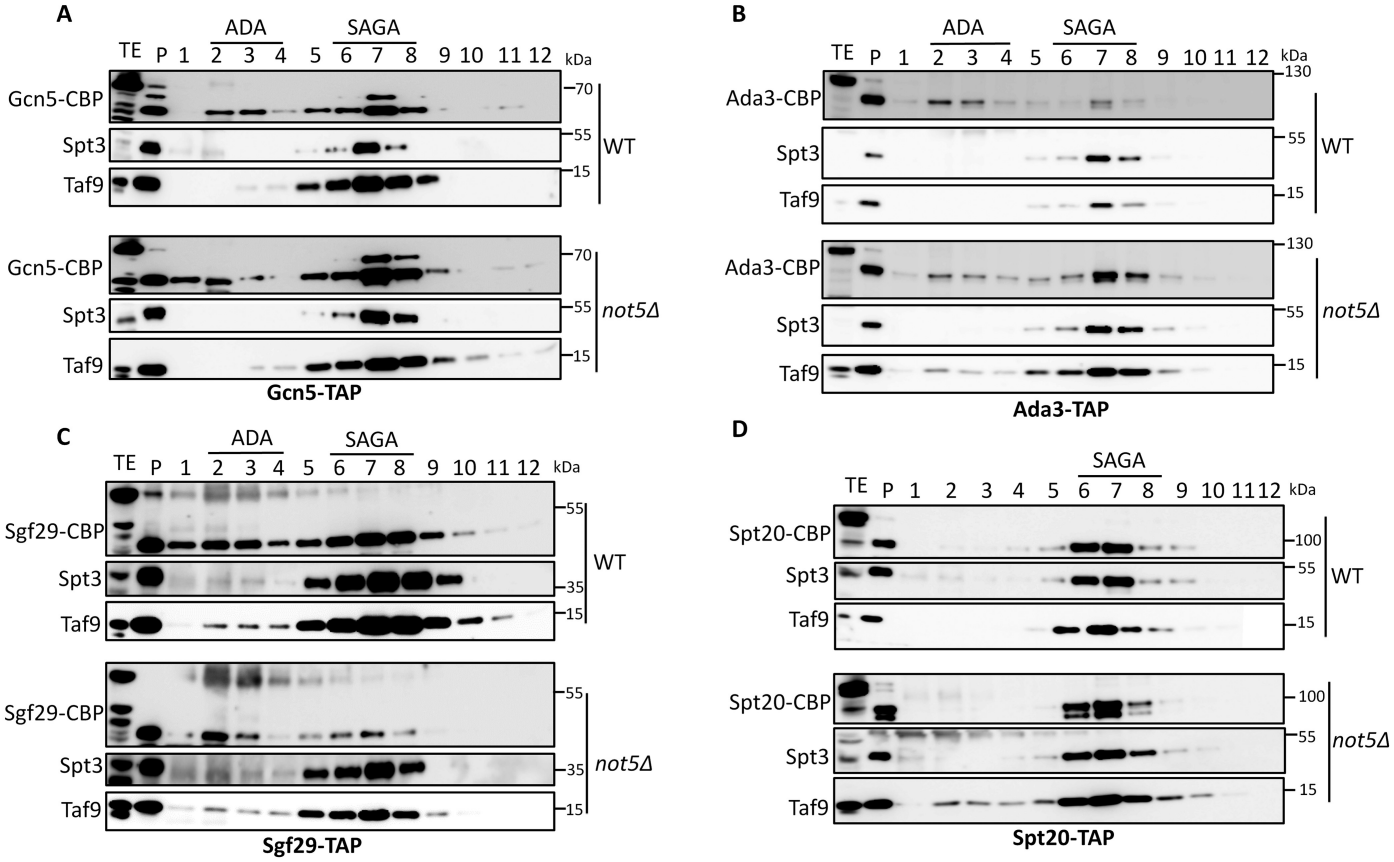
Purification and size fractionation of SAGA subunits. Gcn5-TAP (**A**), Ada3-TAP (**B**), Sgf29-TAP (**C**) and Spt20-TAP (**D**) were purified by a single affinity purification step from WT and *not5Δ* and purified proteins were separated on a 1–20% sucrose gradient. Proteins from the different fractions were TCA precipitated and loaded on SDS-PAGE, and transferred to a membrane that was probed with anti CBP, anti-Taf9 and anti-Spt3 antibodies as indicated. Fraction 1 is the lightest and fraction 12 the heaviest. The position of elution of SAGA and ADA are indicated.

To visualize SAGA complexes better we purified several SAGA subunits, namely Gcn5, Ada3, Sgf29 and Spt20, by a single affinity purification step and separated the purified material on a 1–20% sucrose gradient (Figure 2A–D). All four purified proteins from WT cells were present in the fractions 5–8 of the sucrose gradient, where we could also detect other SAGA subunits, namely Spt3 and Taf9. Gcn5, Ada3 and Sgf29 were additionally detected in fractions 2–5 where Ahc1 eluted (Figure 1C) and corresponding to ADA complexes.

In *not5Δ*, globally more Gcn5 was purified from the mutant despite equal expression compared to the WT. It was detected in fractions 5–8 where SAGA sediments, but also in the two first fractions of the sucrose gradient corresponding to sizes smaller than either ADA or SAGA (Figure 2A). Ada3 was mostly detected in fractions corresponding to SAGA (Figure 2B) whereas Sgf29 was less present in these fractions and consistently less Spt3 and Taf9 were purified with Sgf29 (Figure 2C). Finally, the SAGA-specific subunit Spt20 was detected in fractions 5–8 corresponding to SAGA, but visible both as an intact protein and in a cleaved form (Figure 2D).

These data suggest that the integrity of SAGA is altered: SAGA complexes tend to fall apart upon native gel electrophoresis, less Sgf29 is incorporated into SAGA, and cleaved forms of Spt20 are present in SAGA complexes. Finally Gcn5 is more accessible to purification in *not5Δ*, and Gcn5 is present in complexes that are neither ADA nor SAGA, and smaller.

### Less full-length Spt20 is purified with SAGA subunits in *not5Δ*

**Figure 3.**
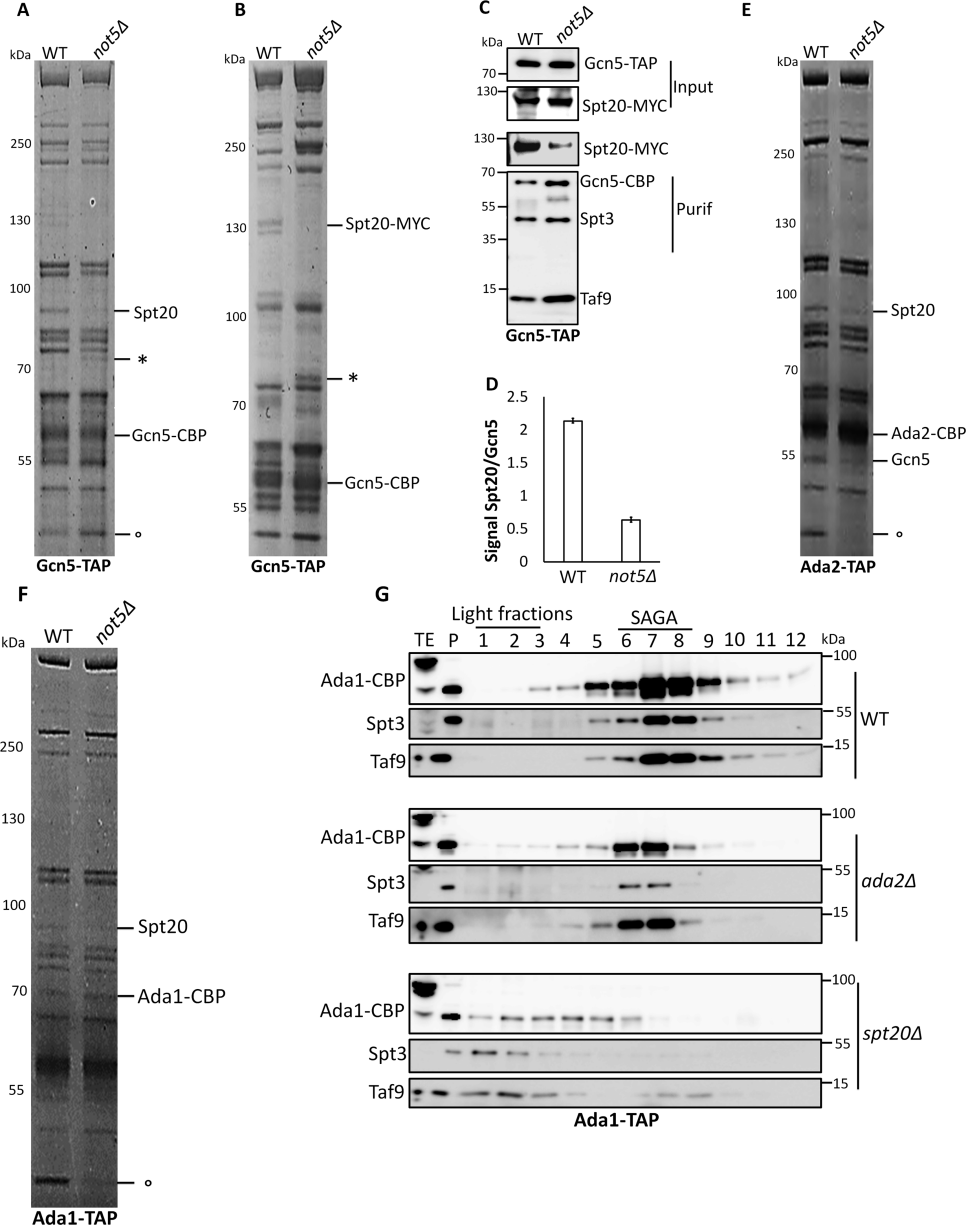
Full-length Spt20 is less purified with various SAGA subunits in *not5Δ* cells. (**A** and **B**) Gcn5-TAP was purified by a single affinity purification step from the indicated cells expressing (A) Spt20 or (B) C-terminally MYC-tagged Spt20. The purified proteins were separated by SDS-PAGE and stained by coomassie. The position of probable truncated forms of tagged or untagged Spt20 is indicated by ‘*’. (**C**) Quantification. Total extract (Input) and the samples from the purification shown in (B) (Purif) were loaded on SDS-PAGE that was transferred and revealed with MYC or CBP antibodies. (**D**) The amount of Spt20-MYC in panel (C) was quantified and normalized to the levels of Gcn5-CBP. (**E**) Ada2-TAP or (**F**) Ada1-TAP were purified by a single affinity purification step and the purified proteins were separated by SDS-PAGE and stained by coomassie. (**G**) Purification of Ada1 from WT, *ada2Δ* or *spt20Δ*. Ada1-TAP was purified by a single affinity purification step from the indicated strains and purified proteins were separated on a 1–20% sucrose gradient. Proteins from the different fractions were TCA precipitated and loaded on SDS-PAGE, and transferred to a membrane that was probed with anti CBP, anti-Taf9 and anti Spt3 antibodies as indicated. Fraction 1 is the lightest and fraction 12 the heaviest. The position of elution of SAGA and ADA are indicated. A non-SAGA co-purifying protein is indicated by ° in panels A, E and F.

SAGA is thought to have a modular structure with Gcn5 being part of the HAT module together with Ada3, Ada2 and Sgf29 (6). None of the HAT subunits besides Gcn5 were detected in 250 kDa complexes on native gels like Gcn5 (Supplementary Figure S2) excluding the idea that the smaller Gcn5 complex corresponds to the HAT module separated from the rest of SAGA. Hence to better understand the defective integrity of Gcn5 complexes in *not5Δ*, we purified Gcn5 from WT and *not5Δ* and separated the purified proteins by SDS-PAGE followed by coomassie staining (Figure 3A). One protein clearly stood out as being significantly reduced in the purification from *not5Δ*, and it was defined by mass spectrometry to be Spt20. To verify this identification, we tagged Spt20, purified Gcn5 again and analyzed the purification on SDS-PAGE followed by coomassie staining (Figure 3B) and western blotting (Figure 3C). This confirmed that a reduced amount of full-length Spt20 co-purified with Gcn5 from *not5Δ* (Figure 3D) despite indistinguishable steady state levels of Spt20 in extracts from both strains (Figure 1D). We also noted evidence for the presence of truncated forms of Spt20 in the purifications of Gcn5 from *not5Δ* (indicated by ‘*’ in Figure 3A and B).

Previous studies have demonstrated that the *in vitro* acetylation function for Gcn5 at nucleosomal substrates requires Ada2 (36) and that it is Ada2 that connects Gcn5 to the rest of the SAGA complex (6). Since we determined that less full-length Spt20 purified with Gcn5 from cells lacking Not5, we tested whether the association of Ada2 with Spt20 was altered in *not5Δ*. Purification of Ada2 by a single affinity purification step from WT and *not5Δ* revealed that, indeed, less full-length Spt20 purified with Ada2 also (Figure 3E). We then purified a SAGA subunit from the SAGA core, namely Ada1, and we also observed less full-length Spt20 in the Ada1 purification (Figure 3F).

Taken together, these observations indicate that less full-length Spt20 purifies not only with HAT module subunits, but generally with SAGA components, in *not5Δ*.

### Spt20 is needed for Ada2 and Gcn5 to incorporate into SAGA complexes

Spt20 is thought to be the subunit that anchors the HAT module to the rest of the SAGA complex (6). If so, then Gcn5 and Ada2 require Spt20 to associate with SAGA. Indeed, Ada2 purified from cells lacking Spt20 associated with Gcn5, but only weakly with the other SAGA subunits (Supplementary Figure S3A, lane 4). Similarly SAGA subunits did not efficiently purify with Gcn5 isolated from *spt20Δ* (Supplementary Figure S3B). Moreover, no SAGA complexes could be purified via Ada1 from cells lacking Spt20 (Figure 3G compare lower three panels with upper three panels). Previous studies have indicated that Sgf73 contributes to the association of Spt20 and Ada2 (6) and we could confirm this (Supplementary Figure S3C). Nevertheless since it was not essential, we focused further on Ada2 and Spt20 only.

We then tested the role of Ada2. Gcn5 and other SAGA subunits purified with Spt20 from *ada2Δ* cells (Supplementary Figure S3A, lanes 2 and 3) and Spt20 purified with Gcn5, but neither Spt3 nor Taf9 purified with Gcn5 from *ada2Δ* (Supplementary Figure S3D). These data indicate that in the absence of Ada2, Spt20 can associate with Gcn5 and Spt20 can associate with other SAGA subunits, but this happens within different Spt20 complexes. Consistently, complexes slightly smaller than SAGA were purified with Ada1 in cells lacking Ada2 (Figure 3G compare three middle panels to upper three panels).

Thus, Gcn5 incorporation into SAGA requires both Ada2 and Spt20, Spt20 is needed for Ada2 to efficiently associate with SAGA subunits. In contrast Gcn5 is not needed for Ada2 and Spt20 to interact (Supplementary Figure S3E).

Our data so far suggest that the integrity of SAGA complexes is defective in *not5Δ* but in apparent contradiction we observed relatively WT amounts of SAGA-sized complexes purified from Gcn5 in *not5Δ* (Figure 2A). We thus did a quantitative mass spectrometry analysis of the Gcn5 purified from WT and *not5Δ*, both the total purified proteins, and the proteins in the SAGA-sized complexes (Supplementary Table S4). Most SAGA subunits were detectable in the total purification of Gcn5 from WT or mutant, and the percentage of Gcn5 spectra relative to other proteins was higher for purification from the mutant than for purification from the WT (0.45% relative to 0.36% for a minimum of 2 peptides and 95% peptide and protein threshold) (Supplementary Table S4). The representation of Gcn5 was also higher in SAGA fractions from *not5Δ* than from those from the WT, whereas many SAGA subunits, in particular Tra1, Spt7, Spt8 and Spt3, instead were less present.

These findings indicated that the SAGA-sized Gcn5 complexes in *not5Δ* were mostly not normal SAGA.

**Figure 4.**
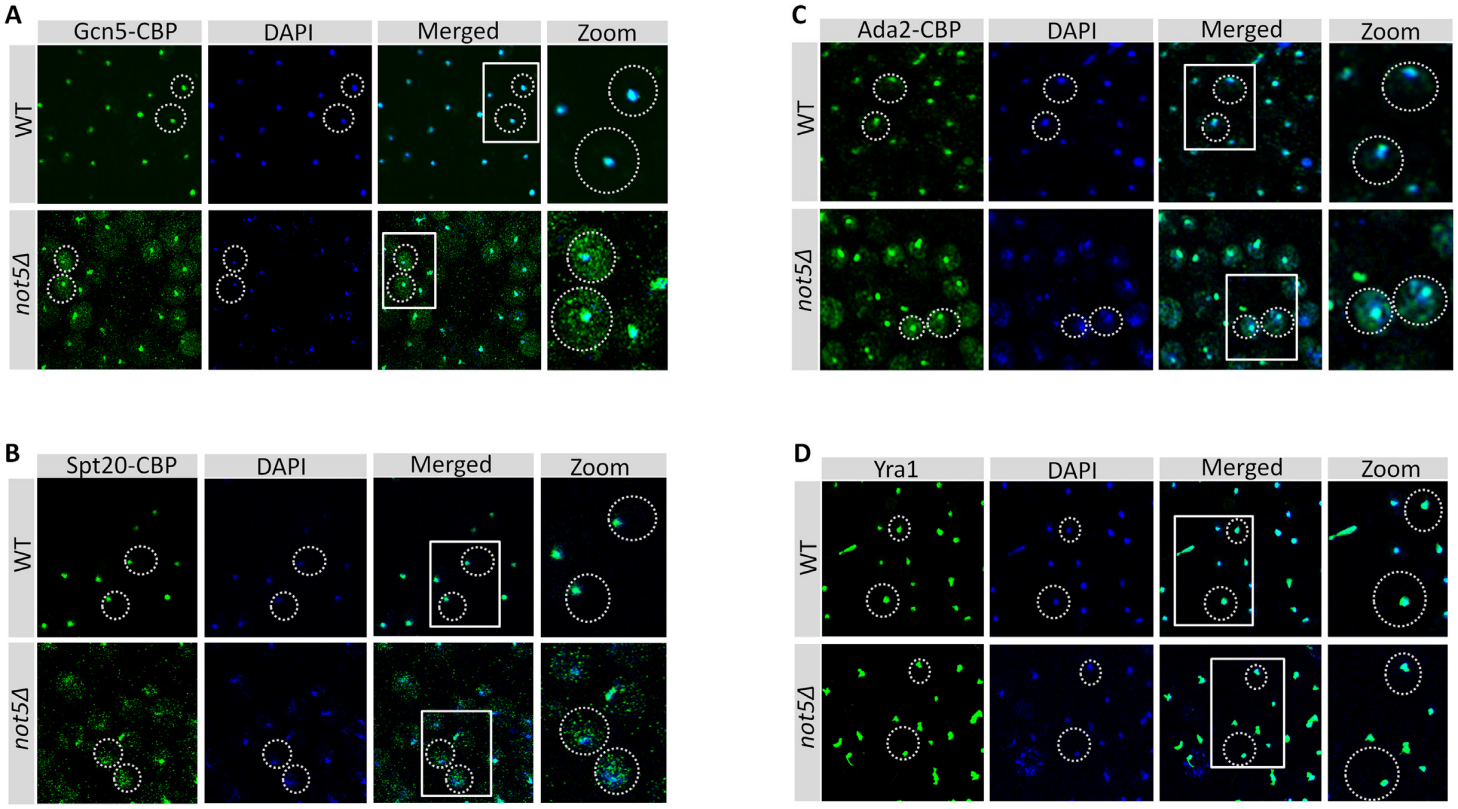
Nuclear localization of Gcn5, Ada2 and Spt20 is defective in *not5Δ*. WT and *not5Δ* cells expressing Gcn5-TAP (**A**), Spt20-TAP (**B**), Ada2-TAP (**C**) were stained with antibodies against CBP (left panels), DAPI (middle panels) and the images were merged (right panels). (**D**) As a control, the Yra1 protein was stained with Yra1 antibodies (left panel), DAPI (middle panels) and the images were merged (right panels). For (**A–D**), on the far right is a zoom of the boxed regions in the right panels. More than 200 cells were scored for each case. Defective nuclear staining of the SAGA subunits in the mutant was observed in 100% of the scored cells.

To determine whether these defects of SAGA integrity in *not5Δ* extracts *in vitro* correlated with *in vivo* defects, we used immunofluorescence and followed Gcn5, Spt20 and Ada2. Gcn5 showed an exclusively nuclear localization in the WT, but in *not5Δ* Gcn5 was additionally visible as dots all over the cytoplasm (Figure 4A). The same was observed for both Spt20 (Figure 4B) and Ada2 (Figure 4C). To know if this was specific to these SAGA subunits we looked at another nuclear protein, Yra1 and it showed normal nuclear localization in cells lacking Not5 (Figure 4D).

Hence the defective integrity of SAGA detected *in vitro* correlated with a defect in nuclear localization of SAGA subunits *in vivo*.

### Ada2 presence at polysomes translating *SPT20* mRNA requires Not5

**Figure 5.**
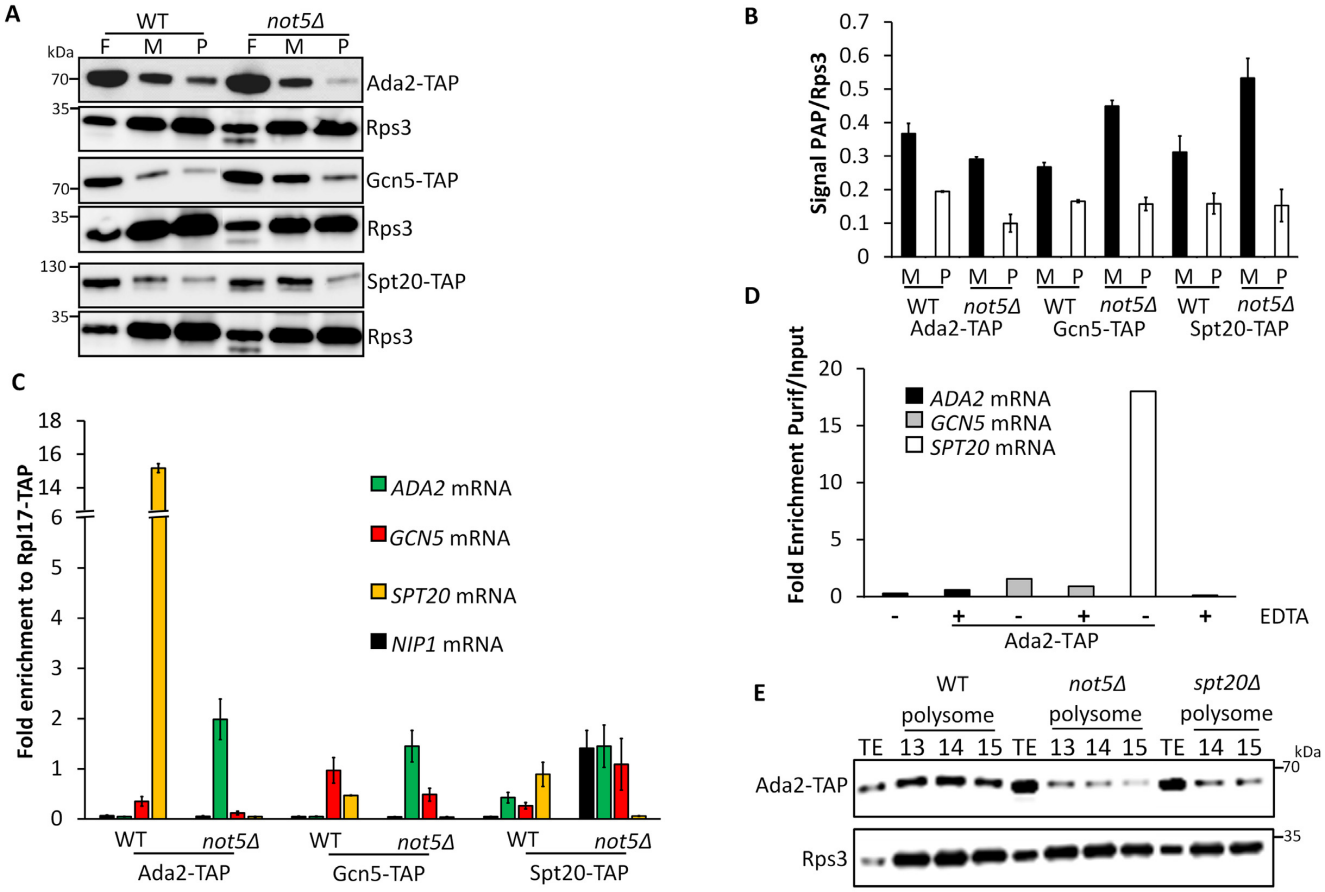
Ada2 is present at polysomes producing Spt20. (**A**) Analysis of Ada2, Gcn5 and Spt20 in sucrose gradient fractions. Total protein extracts from WT or *not5Δ* cells expressing Ada2-TAP, Gcn5-TAP or Spt20-TAP were separated on a 7–47% sucrose gradient (Supplementary Figure S3A). Proteins from fractions corresponding to free RNAs (F), monosomes (M) or polysomes (P) were TCA precipitated and loaded on SDS-PAGE followed by western blotting with PAP antibodies or Rps3 antibodies to normalize for ribosome content. (**B**) Quantification of the results from panel A. The signal in panel (A) for PAP was expressed relative to the signal for Rps3 in the monosome and polysome fractions. (**C**) RIP of Ada2, Gcn5 and Spt20 from polysome fractions. Extracts from WT and *not5Δ* cells expressing Ada2-TAP, Gcn5-TAP, Spt20-TAP and Rpl17-TAP were separated on a 7–47% sucrose gradient. Polysome fractions were pooled and loaded on IgG sepharose beads. The bound protein was eluted by TEV cleavage. RNA was extracted from the eluate and the levels of *ADA2, GCN5, SPT20* or *NIP1* mRNAs were evaluated by RT-qPCR. The amount of mRNA in the RIPs from SAGA subunits was expressed relative to the amount of the same mRNA RIP-ed by Rpl17. (**D**) RIP of Ada2 from total extracts. Total protein extracts from cells expressing Ada2-TAP and treated or not with EDTA were loaded on IgG sepharose beads. Ada2-CBP was eluted by TEV cleavage. RNA was extracted from the eluate (Purif) and the levels of *ADA2, GCN5* and *SPT20* mRNAs were evaluated by RT-qPCR and expressed relative to the amount in the total extract (Input). (**E**) Presence of Ada2 in polysomes of WT, *not5Δ* and *spt20Δ*. Total extracts from WT, *not5Δ* and *spt20Δ* expressing Ada2-TAP were separated on sucrose gradients 7–47%. The amount of Ada2 in the total extract (TE) and polysome fractions (13–15) was analyzed by western blotting.

One possible explanation for the SAGA integrity defects described above is an assembly defect. Since it has been reported that assembly of multi-subunit complexes occurs in several instances co-translationally (13) we considered that this might be the case for SAGA. We analyzed the distribution of Gcn5, Ada2 and Spt20 across a sucrose gradient. We observed that all three proteins co-sedimented in polysome fractions (Supplementary Figure S4A). Quantification of SAGA subunits present in monosome and polysome fractions in WT and *not5Δ* revealed that lower amounts of Ada2 was present in both monosome and polysome fractions in *not5Δ* (Figure 5A and B). Gcn5 and Spt20 were present at similar levels in polysomes from *not5Δ* and WT, but they accumulated in monosomes in the mutant. These observations were compatible with a co-translational assembly of these SAGA subunits that could be altered in the absence of Not5. To determine which mRNAs were being translated in the polysomes in which Gcn5, Ada2 and Spt20 were present, we immunoprecipitated these SAGA subunits from WT and *not5Δ* polysome fractions and analyzed the immunoprecipitates for the presence of specific mRNAs (RNA immunoprecipitations or RIPs). RIPs were also performed from strains expressing a tagged version of the accessible Rpl17 ribosomal subunit to normalize the SAGA RIPs taking into account the global level of each mRNA in polysomes (mRNA translatability). The *SPT20* mRNA was enriched in the Ada2 RIP from the WT but not from *not5Δ* (Figure 5C). The other mRNAs were not significantly enriched in any of the RIPs. A different way to assess whether Ada2 is associated with *SPT20* mRNA in polysomes is to immunoprecipitate Ada2 from total extracts in the presence or absence of EDTA, which disrupts polysomes (Supplementary Figure S4B). We observed that indeed, the presence of *SPT20* mRNA in the Ada2 RIP from total extracts required polysome integrity (Figure 5D). Finally, we wanted to determine whether Ada2 was present in polysomes translating proteins other than Spt20. We fractionated extracts from cells lacking *SPT20* in a sucrose gradient and followed Ada2. The presence of Ada2 in polysomes was very much reduced in *spt20Δ* polysomes, as it was in polysomes from *not5Δ* (Figure 5E).

This reduced presence of Ada2 in polysomes in the absence of *SPT20* mRNA indicated that Ada2 was mainly or only present at polysomes translating *SPT20*. Moreover our results indicated that the presence of Ada2 at this site requires Not5.

### Ccr4–Not is present in polysomes translating *ADA2* and *SPT20*

**Figure 6.**
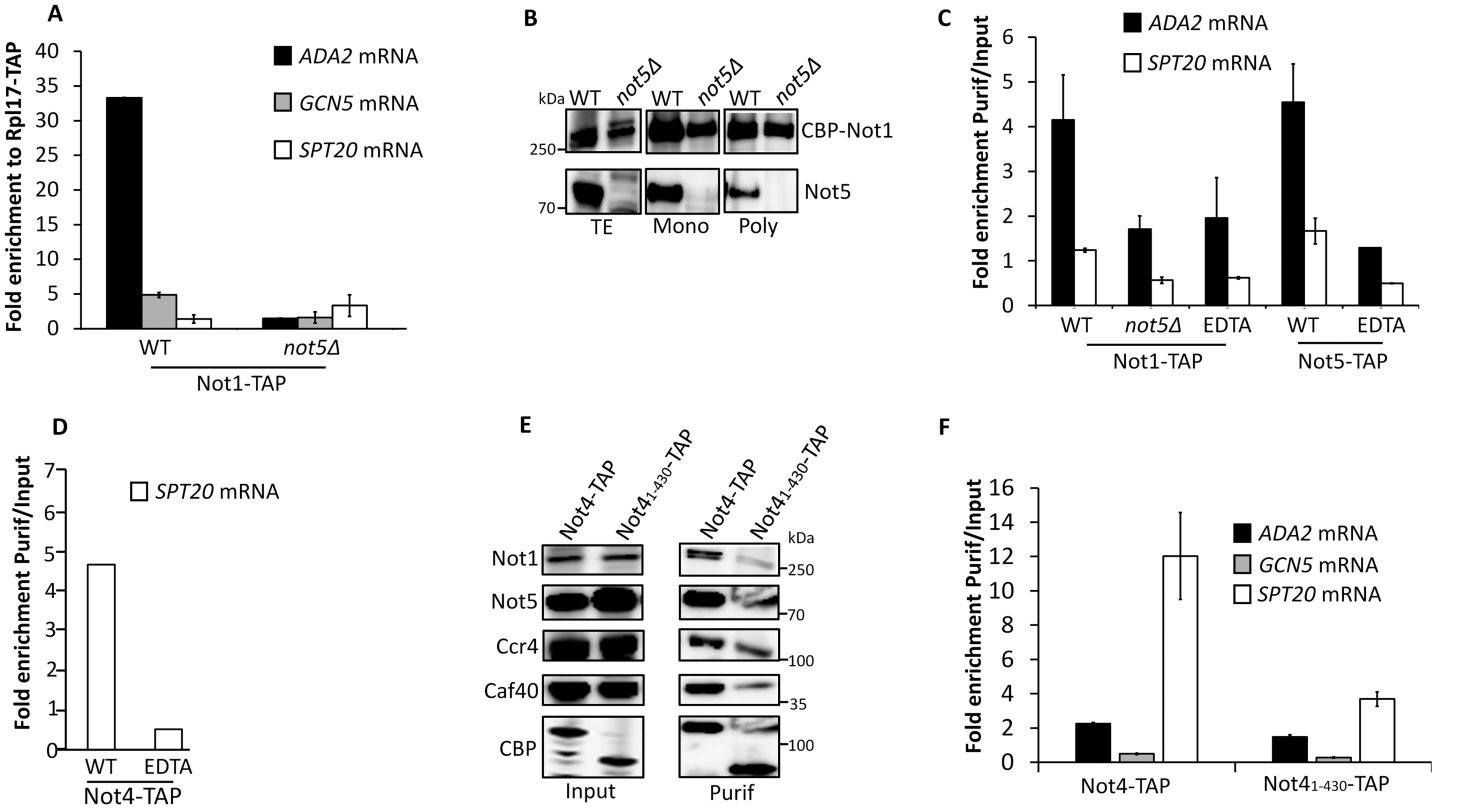
Ccr4–Not is at polysomes producing SAGA subunits. (**A**) RIP of Not1 from polysomes. Total protein extracts from WT or *not5Δ* cells expressing Not1-TAP were separated on a 7–47% sucrose gradient. Polysome fractions were loaded on IgG sepharose beads and the bound protein was eluted by TEV cleavage. RNA was extracted from the eluate and the levels of *ADA2, GCN5* or *SPT20* mRNAs were evaluated by RT-qPCR, and expressed relative to the amount of the same mRNA RIPed by the ribosomal protein Rpl17-TAP. (**B**) IP of Not1 from total extracts, monosomes and polysomes in WT and *not5Δ*. Total extracts (TE) and fractions corresponding to monosomes (9-10, Mono) or polysomes (13-15, Poly), obtained by 7–47% sucrose gradient centrifugation of TAP-tagged-Not1 extracts from WT and *not5Δ* were analyzed by western blotting with antibodies against CBP and Not5. (**C**) RIP of Not1 and Not5 from total extracts. Total protein extracts from WT and *not5Δ* cells expressing Not1-TAP or WT cells expressing Not5-TAP and treated or not with EDTA were loaded on IgG sepharose beads. Not1-CBP or Not5-CBP were eluted by TEV cleavage. RNA was extracted from the eluate and the levels of *ADA2* and *SPT20* mRNAs were evaluated by RT-qPCR and expressed relative to the amount in the total extract. (**D**) RIP of Not4 from total extracts. Total protein extracts from cells expressing Not4-TAP and treated or not with EDTA were loaded on IgG sepharose beads. Not4-CBP was eluted by TEV cleavage. RNA was extracted from the eluate (Purif) and the levels of *SPT20* mRNA were evaluated by RT-qPCR and expressed relative to the amount in the total extract (Input). (**E**) IP of Not4 and truncated Not4. The same experiment as in panel D but with a truncated Not4_1-430_-TAP was performed. The total extracts (Input) and the Not4-CBP and Not4_1-430_-CBP eluates (Purif) were tested by western blotting for the levels of the different Ccr4–Not subunits. (**F**) RIP of Not4 and truncated Not4 from total extracts. The levels of *ADA2, GCN5* and *SPT20* mRNAs in the IP were evaluated and expressed relative to the level in total extracts.

Several studies have reported that Ccr4–Not subunits are present at polysomes while others have described that the association of the scaffold subunit Not1 with specific mRNAs is altered in the absence of Not5 (38–41). We thus determined whether Not1 was present in polysomes translating *ADA2, GCN5* or *SPT20*. We performed Not1 RIPs from polysome fractions in WT or *not5Δ* cells. As before, we normalized the results according to the Rpl17 RIP from polysomes of the same strains used as a control for global presence of the mRNAs in polysomes (Figure 6A). The *ADA2* mRNA was highly enriched in the Not1 RIPs from the WT and this was reduced in *not5Δ*. This was specific because the global presence of Not1 in polysomes was not changed in the absence of Not5 (Figure 6B). The enrichment of *SPT20* in the Not1 RIPs from polysomes was not significant whereas some enrichment of the *GCN5* mRNA was detected (Figure 6A). As before for Ada2, we also performed Not1 and Not5 RIPs from total extracts treated or non-treated with EDTA. We detected enrichment of *ADA2* mRNA in Not1 and Not5 RIPs from WT cells that were dependent upon polysome integrity but we observed no Not1 RIP of *ADA2* mRNA from total extracts of *not5Δ* (Figure 6C). The RIP results presented above determine the presence of the Ccr4–Not complex at polysomes producing Ada2, but not Spt20. However negative RIP experiments cannot always distinguish between the absence of an interaction and inaccessibility of the epitope that is targeted for immunoprecipitation. Not4 is another subunit of the Ccr4–Not complex that is present at polysomes and has been connected to co-translational quality control (39,42,43). It harbors an RNA recognition motif (44,45) and like Not5 is required for global acetylation levels (24). We did RIPs via Not4 in the presence or absence of EDTA and determined that *SPT20* mRNA was immunoprecipitated with Not4 in a polysome integrity-dependent manner (Figure 6D). A compelling open question about the Ccr4–Not complex is whether it works as a unique entity *in vivo*, or whether the subunits may also work separately. This is particularly true for Not4 that, while it is co-purified with the other Ccr4–Not subunits in yeast, is not a stable subunit of the complex in higher eukaryotes. The domain of Not4 that interacts with Not1 has been determined in yeast (46) and by structure resolution (47). It involves a domain in the C-terminus of Not4. The interaction between Not4 and Not1 is lost when cells express a C-terminal truncated version of Not4 beyond residue 430. We wanted to determine whether the presence of Not4 at polysomes producing Spt20 required its association with Not1, since Not1 itself could not RIP *SPT20* mRNA. We performed a new RIP with the truncated Not4 that was as well expressed as the full-length protein. It was as efficiently immunoprecipitated as full length Not4, but as expected did not co-immunoprecipitate the other Ccr4–Not subunits (Figure 6E). *SPT20* mRNA was much less enriched in the RIP with the truncated Not4. We additionally weakly detected *ADA2* mRNA in the Not4 RIP and this was also reduced in the RIP with the truncated Not4 (Figure 6F). *GCN5* mRNA instead was not detected in the Not4 RIP. Taken together these finding indicates that Not1 is present at polysomes translating Ada2 and Spt20 and that Not5 is needed for Not1 presence at *ADA2* polysomes. Not4 in turn is present in polysomes producing Spt20 and this requires its interaction with Not1.

### Tethering of SAGA mRNAs together does not require Not5

**Figure 7.**
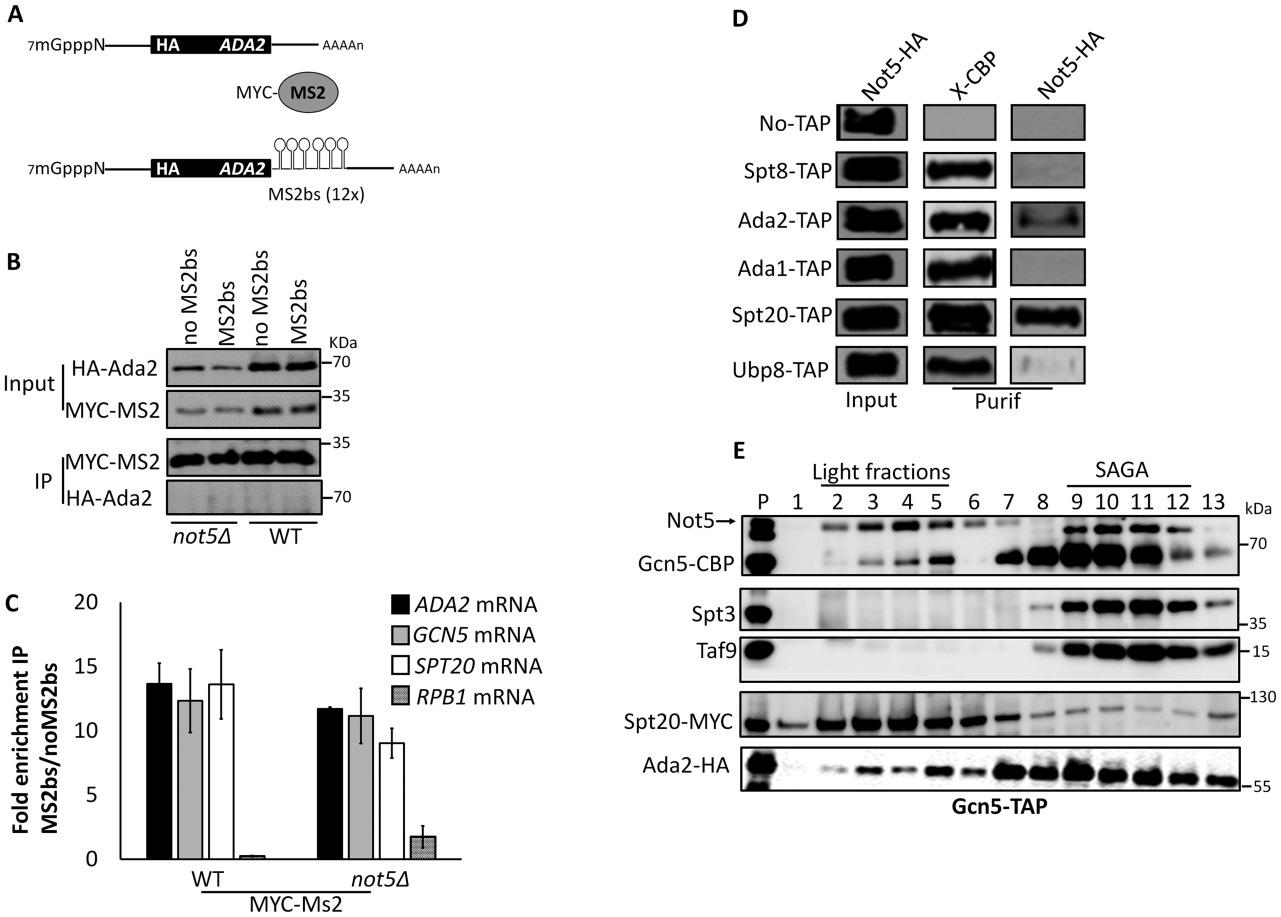
Some of the SAGA mRNAs are tethered together. (**A**) Cartoon of the *ADA2* reporter mRNAs analyzed. Both reporters were expressed under the control of the *SPT3* promoter and *CYC1* terminator and have also been engineered to express an HA tag at the N-terminus of Ada2. We verified that such an *ADA2* reporter complements the deletion of *ADA2*. The second reporter has 12 MS2 binding stem-loops (MS2bs) in its 3΄ UTR. (**B**) IP of MS2 from WT and *not5Δ*. WT and *not5Δ* cells were transformed with plasmids expressing the mRNAs depicted in (A). They were additionally transformed with a plasmid expressing MYC-MS2. MS2 was immunoprecipitated and the levels of HA-Ada2, MYC-MS2 in the input and in the precipitate (beads) were evaluated. (**C**) RIP of MS2. RNA was extracted from the immunoprecipitate and the levels of *SPT20, GCN5, ADA2* and *RPB1* mRNAs were evaluated by RT-qPCR and quantified relative to the amount from the immunoprecipitate of the control. (**D**) IP of Not5 with certain SAGA subunits. Cells expressing the indicated Tap-tagged proteins and HA-tagged Not5, or untagged cells (no TAP) as a control, were loaded on IgG sepharose beads. The CBP-tagged proteins were eluted by TEV cleavage and the presence of the eluted protein (X-CBP) and Not5-HA was evaluated in the total extract (Input) and eluate (Purif) by western blotting with CBP or HA antibodies as indicated. The complete blots of this experiment are shown in Supplementary Figure S8. (**E**) Gcn5-TAP was purified from cells expressing Spt20-MYC and Ada2-HA by single affinity and then loaded on a 1–20% sucrose gradient. The fractions were tested by western blotting for the presence of the indicated proteins. Of the shown fractions, 1 is the lightest and 13 the heaviest.

The results presented so far indicate that Not5-dependent Not1 association with *ADA2* mRNA correlates with the need of Not5 for the presence of Ada2 at polysomes producing Spt20, for functional interaction of Ada2 and Spt20 and for functional integration of Gcn5 into SAGA. However, the exact role of Not5 in this mechanism is unclear. One possibility is that polysomes translating the *ADA2* and *SPT20* mRNAs co-localize such that Ada2 can be present during production of Spt20. Not5-dependent association of Not1 with *ADA2* mRNA might be necessary for these mRNAs to co-localize. To test this hypothesis, we relied on the MS2 bacteriophage system. We inserted an MS2 binding sequence (MS2bs) in the 3΄untranslated region of *ADA2* mRNA carried on a plasmid that we first verified functionally complemented the deletion of *ADA2*. Plasmids carrying this construct with or without the MS2bs loop were transformed in WT and *not5Δ* cells in which we expressed a MYC-tagged MS2 coat protein (Figure 7A). We immunoprecipitated MYC-MS2 and tested the immunoprecipitate for *ADA2, GCN5* and *SPT20* mRNAs, as well as for the unrelated *RPB1* mRNA encoding the largest subunit of RNAPII. We determined that *ADA2* carrying the MS2bs was specifically immunoprecipitated by the MS2 protein. In addition, *SPT20* and *GCN5* mRNAs, but not *RPB1*, were enriched in the immunoprecipitate (Figure 7B and C). The result was similar for WT and *not5Δ* cells. Hence *ADA2, SPT20* and *GCN5* mRNAs are tethered together, but this does not need Not5. To determine whether the mRNAs were present at the sites of production of the proteins, we repeated the experiment, but immunoprecipitated MS2 from polysome fractions after separation of the total extracts on a sucrose gradient. The result was very similar (Supplementary Figure S5) indicating that the SAGA mRNAs are tethered together at their site of translation.

Since Not5 is not needed to tether SAGA mRNAs together, it might instead be necessary to retain newly produced Ada2 at the polysomes where Spt20 is being synthesized. The presence of Not5 at the site of Spt20 production is supported by our observation that Not5 co-immunoprecipitated specifically with Spt20 and to a lesser extent with Ada2, but not for instance with other SAGA subunits such as Spt8 or Ada1 (Figure 7D). Taken together these findings are consistent with a model in which the Not proteins might interact with the newly produced proteins, Ada2 and/or Spt20, to keep them tethered at sites of production and/or promote their productive interaction. In the absence of Not5, the proteins apparently escape from the site of production before productive assembly and tend to aggregate (Ada2, Spt20 and Gcn5).

### Tdh3 is needed for SAGA assembly

**Figure 8.**
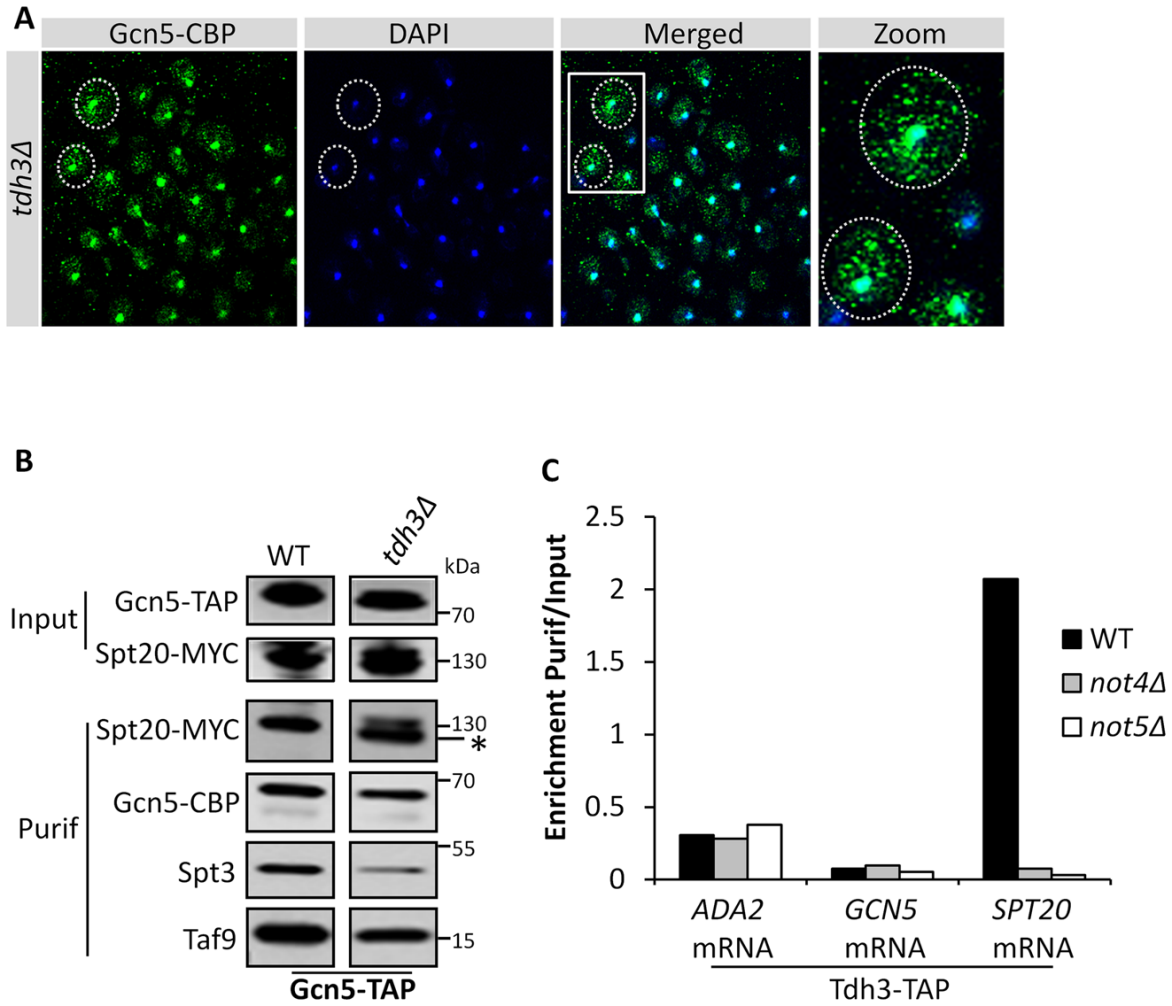
Tdh3 is required for Spt20 integrity and SAGA assembly. (**A**) Nuclear localization of Gcn5 needs Tdh3. WT and *tdh3Δ* cells expressing Gcn5-TAP were analyzed by immunofluorescence with antibodies against CBP (left panel), DAPI (middle panel) and the staining was merged (right panel). The boxed areas in the right panel are shown enlarged on the far right. (**B**) Spt20 is cleaved in the IP of Gcn5 from *tdh3Δ*. Extracts from WT and *tdh3Δ* cells expressing Gcn5-TAP and Spt20-MYC were loaded on IgG sepharose. Gcn5-CBP was eluted by TEV cleavage. The total extract (Input) and eluate (Purif) were analyzed by western blotting for the presence of the indicated proteins. ‘*’ indicates a cleaved form of Spt20. (**C**) RIP of Tdh3 from total extracts. Extracts from WT, *not4Δ* and *not5Δ* cells expressing Tdh3-TAP were loaded on IgG sepharose. Tdh3-CBP was eluted by TEV cleavage. RNA was extracted from the eluate and the levels of *SPT20, GCN5* and *ADA2* mRNAs were evaluated by RT-qPCR and expressed relative to the amount in the total extract.

In our experiments we noticed that truncated forms of Spt20 were present in *not5Δ*, both N-terminally truncated forms (indicated by ‘*’ in Figure 3A) and C-terminally truncated forms of Spt20 (associated with Gcn5 and indicated by ‘*’ in Figure 3A and B). If this was strictly due to the absence of Ada2 during production of Spt20, this same phenotype of Spt20 truncation should be visible in cells lacking Ada2. However, we did not detect any truncation of Spt20 in cells lacking Ada2 (see Supplementary Figure S3A). Hence the role of Not5 during production of Spt20 might extend beyond ensuring that Ada2 is present. A hint came from the purification of Ada2, in which we determined that a small protein not compatible with the size of any known SAGA subunit was present in the purification from the WT but not from *not5Δ* (Figure 3E indicated by °). A similar protein was detected in the purification of Gcn5 from the WT and the mutant (see in Figure 3A). Fractionation of Gcn5 co-purifying proteins on a sucrose gradient showed that this protein is not present in SAGA complexes, but instead is present in fractions containing small Gcn5 complexes (Supplementary Figure S6A). We identified this protein by mass spectrometry to be Tdh3, namely glyceraldyde-3-phosphate-dehydrogenase, an enzyme of the glycolytic pathway known to function as a moonlighting protein. Tagging and deletion of Tdh3 in cells from which we purified Ada2 confirmed that the protein that co-purified with Ada2 in WT cells but not mutant cells is Tdh3 (Supplementary Figure S6B). To determine whether Not5-dependent co-purification of Tdh3 with Ada2 had any functional role for Gcn5 integration into SAGA, we tested extracts from *tdh3Δ* cells on native gels. We observed that Gcn5 was present in small complexes as in *not5Δ* (Supplementary Figure S6C). Moreover, Gcn5 was also detected in cytoplasmic speckles in the cytoplasm of cells lacking Tdh3 as in cells lacking Not5 (Figure 8A). Finally, we observed some N-terminal cleavage of Spt20 co-purified with Gcn5 from cells lacking *tdh3Δ* (Figure 8B). We performed RIP experiments with Tdh3 to determine whether Tdh3 was present at the polysomes producing Spt20. Indeed, *SPT20* mRNA was immunoprecipitated with Tdh3 and this required both Not4 and Not5 (Figure 8C). Together these results indicate that Tdh3 is present at polysomes producing Spt20, in a Not4 and Not5-dependent manner and it co-purifies with Ada2 in a Not5-dependent manner. This is important for Spt20 integrity, and contributes to proper incorporation of Gcn5 into SAGA. One possible explanation for our observations could be that the Not proteins tether Tdh3 to polysomes producing Ada2 and Spt20. Tdh3 is a very abundant protein and it is difficult to obtain very convincing co-immunoprecipitation results with clear negative controls. Hence we used the yeast two hybrid assay that has already been successfully used for Tdh3 (35) and tested the interaction of Tdh3 and Not1. We obtained a clear positive signal for an interaction between Not1 and Tdh3 (Supplementary Figure S7). Hence Not1 interacts with Tdh3 and can tether Tdh3 to polysomes producing Spt20. In turn Tdh3 protects newly produced Spt20 from cleavage allowing Spt20 to associate with Ada2 and contributing to productive association of Gcn5 into SAGA.

## DISCUSSION

**Figure 9.**
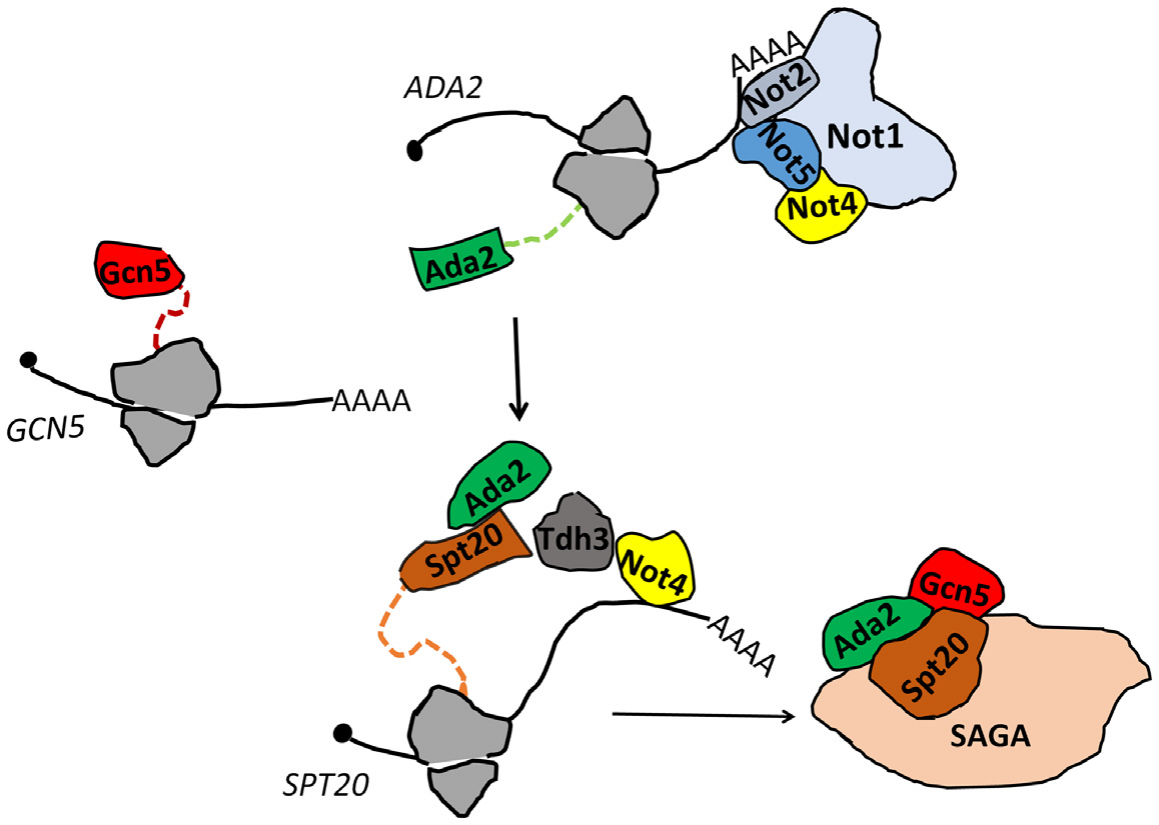
Model for the importance of the Not proteins and Tdh3 during co-translational assembly of SAGA. The *ADA2, SPT20* and *GCN5* mRNAs are tethered at their site of translation. The Ada2 protein is present at the site of Spt20 production. This needs expression of Not5. Not1 is present at the site where *ADA2* is translated in a Not5-dependent manner. Not4 binds *SPT20* mRNA if it can associate with Not1. Tdh3 associates with *SPT20* mRNA if Not4 and Not5 are expressed. Tdh3 and Not5 are needed for integrity of Spt20. Appropriate nuclear localization of Gcn5, Ada2 and Spt20 needs Not5, and that of Gcn5 needs Tdh3. We propose that association of Not1 with *ADA2* mRNA in polysomes needs Not5, and allows Not1 to promote association of Ada2, Not4 and Tdh3 with *SPT20* mRNA in polysomes. This ensures appropriate association of Ada2 with Spt20 and integrity of Spt20, and finally interaction with newly produced Gcn5. In turn these co-translational events are the ones that ensure optimal integrity of SAGA. Otherwise the subunits are less faithfully incorporated into SAGA and will aggregate in the cytoplasm.

In this work we determined that the Not5 subunit of the Ccr4–Not complex is important for integrity of the ADA and SAGA complexes. For SAGA we show that this is because Not5 is necessary for co-translational association of Ada2 with Spt20. We determine that the *ADA2, SPT20* and *GCN5* mRNAs are tethered together and that Not5 is needed for the presence of Not1 at this site of translation to retain Ada2, such that it can properly associate with newly produced Spt20. Finally we demonstrate Not4- and Not5-dependent presence of Tdh3, encoding the moonlighting protein glyeraldehyde-3-phosphate dehydrogenase, at the site of Spt20 production. We show that Tdh3 is also necessary for functional assembly of SAGA (see model on Figure 9). The relevance of our findings in vivo is confirmed by mis-localization of Ada2, Gcn5 and Spt20 in cytoplasmic speckles of cells lacking either Not5 or Tdh3.

Our work brings new understanding about SAGA assembly *in vivo*, since we show that *GCN5, ADA2* and *SPT20* mRNAs are tethered together and that Ada2 and Spt20 must be co-translationally assembled for integrity of SAGA. We determine that complexes of Gcn5 or Ada2 with Spt20 can form in cells lacking Not5, probably post-translationally, but we demonstrate that this occurs with compromised integrity of Spt20, and hence SAGA.

Besides establishing that a core set of SAGA subunits must assemble co-translationally to ensure SAGA integrity, our work also defines that a prototypical moonlighting protein, Tdh3, must be present at this site of SAGA co-translational assembly to ensure Spt20 integrity and hence functions in this case as a molecular chaperone.

Several studies have previously connected the Ccr4–Not complex to SAGA, however no mechanism has emerged to define the exact connection between the two protein complexes. In this work we took a systematic approach to revisit the link between Ccr4–Not and SAGA. We focused on the Not5 subunit of the Ccr4–Not complex, for which there was evidence that it was important for SAGA function. However we have observed similar phenotypes in cells lacking Not2 known to function together with Not5 in a heterodimer (40,48–50). We observe to a lesser extent the same phenotype in cells lacking Not4, but not in other mutants of the Ccr4–Not complex that we tested (*not3Δ, caf1Δ, ccr4Δ, caf130Δ*, our unpublished results). This is consistent with previous studies connecting mostly Not4 and Not5 to levels of global acetylation, and with a former study reporting an interaction between Ada2 and Not2 (21).

We determine that Not5 is needed to ensure Not1 association with SAGA mRNAs. Many Ccr4–Not subunits are less expressed in cells lacking Not5 (51) so Not5 might not be directly responsible for tethering Not1 to these SAGA mRNAs. However our results suggest that Not5 might serve an additional more direct role at the site of Spt20 production. Indeed, we determine that Not5 interacts with Ada2 and Spt20 and hence might be contributing to their association.

Our findings add SAGA to a growing list of protein complexes for which we have now accumulated data showing that they need the Not proteins for complex integrity. One example is the proteasome, for which our laboratory had shown that its functional integrity depends upon the Not4 subunit of Ccr4–Not, at least in part because it is needed for effective interaction of the proteasome chaperone Ecm29 with proteasome subunits (46). Another example is the RNAPII complex for which another study from our group has indicated that Not5 is needed for the co-translational interaction of its largest subunit Rpb1, with its chaperone R2TP, to form a soluble assembly-competent entity (38). A common theme between all of these examples is the need for the Not proteins to ensure presence of chaperones or assembly factors at sites of newly produced proteins. The different studies reveal that the components that need to be tethered at sites of protein synthesis are diverse, Ecm29 for the proteasome, R2TP is necessary for Rpb1 and both Tdh3 and Ada2 during production of Spt20. Finally, it is interesting to note that in this work we studied the molecular explanation for the defect of SAGA integrity in *not5Δ*, but we also demonstrated that the integrity of ADA was compromised in *not5Δ*. It could be that ADA integrity requires the Ccr4–Not complex for a similar co-translational assembly mechanism.

The function of the NOT module of the Ccr4–Not complex has remained elusive so far. Its structural characterization has determined that it offers a large number of interaction surfaces (40,50) and this would agree with a model such as the one we propose in this work. In our study we focus on the SAGA complex, but a similar mechanism might concern many more protein complexes. Indeed, co-translational assembly is thought to be widespread (13) and we have already connected the Not proteins in previous studies to functional integrity of at least two other complexes, namely RNAPII and the proteasome (38,46). The importance of the Not module for co-translational assembly of a diversity of cellular protein complexes certainly would explain the very essential nature of this module in animals and in yeast.

## Supplementary Material

Supplementary Data
