# Not5-dependent co-translational assembly of Ada2 and Spt20 is essential for functional integrity of SAGA

**DOI:** 10.1093/nar/gkx447

**Published:** 2017-05-10

**Authors:** Sari Kassem, Zoltan Villanyi, Martine A. Collart

**Affiliations:** Department of Microbiology and Molecular Medicine, Faculty of Medicine, Institute of Genetics and Genomics Geneva, University of Geneva, Rue Michel-Servet 1, 1211 Geneva 4, Switzerland

*Nucleic Acids Res* (2017) 45 (3): 1186–1199. doi: 10.1093/nar/gkw1059

An error in the above paper has now been corrected. The first paragraph of the Materials and Methods section previously referred to pGREG516, and this has now been corrected to pGREG535. The authors apologise for the error.

