# Supplementary material for: Not5-dependent co-translational assembly of Ada2 and Spt20 is essential for functional integrity of SAGA
Source: Nucleic Acids Res. 2016 Nov 29;45(3):1186–99. doi: 10.1093/nar/gkw1059 (PMC5388395; doi:10.1093/nar/gkw1059)
Supplement: Supplementary Data [file gkw1059_Supp.zip › nar-01067-v-2016-File011.pdf]

Supplementary information file

Supplementary figure legends

Supplementary Figure S1. Analysis of SAGA subunit stability in wild type and *not5Δ*. (**A**) Wild type (WT) and *not5Δ* cells expressing the indicated proteins were grown to exponential phase (time 0) and then collected at the different time points indicated (0.5, 1, 2, and 4 hr) after treatment with cycloheximide (+CHX) or without addition of cycloheximide (-CHX). Total protein extracts from these aliquots were separated on SDS-PAGE and transferred to membranes that were revealed with PAP antibodies except Ada1-Tap that was measured after 2, 4 and 8 h treatment with cycloheximide (+CHX) or without addition of cycloheximide (-CHX). Gcn5-MYC was measured with MYC antibodies. (**B**) Ponceau staining of the membranes prior to the western blot analysis of **Figure 1D** is provided to show equal loading.

Supplementary Figure S2. Integrity of complexes containing SAGA subunits were compared between wild type and *not5Δ*. (A) The same amount of total protein extracts from wild type (WT) and *not5Δ* cells expressing Ada3-TAP, Ada2-TAP, Sgf29-TAP, Ada1-TAP, Spt3-TAP or Spt7-TAP were separated by Native-PAGE (lower panels) to see protein complexes or SDS-PAGE (upper panels) to see equal loading, and transferred to membranes that were revealed with PAP antibodies. Molecular weight markers are indicated on the right.

Supplementary Figure S3. Ada2 connects Gcn5 to SAGA via Spt20. (A) Extracts from wild-type (WT), *not5Δ* and *ada2Δ* cells expressing Spt20-TAP (lanes 1-3) or *spt20Δ* cells expressing Ada2-TAP (lane 4), all expressing also Gcn5-MYC were loaded on IgG sepharose beads and the tagged proteins were eluted by TEV cleavage. The eluted proteins (Purif) and

the total extract (Input) were loaded on SDS-PAGE and transferred to membranes for western blotting with antibodies specific for the indicated proteins. * indicates a cleaved form of Spt20. **(B)** Same as in panel (**A**) with *spt20Δ* cells expressing Gcn5-TAP and carrying or not a plasmid expressing HA-Spt20. **(C)** Same as in (**A**) with wild type and *sgf73Δ* cells expressing Ada2-TAP and expressing Spt20-HA. **(D)** Same as in panel **(A)** with *ada2Δ* cells expressing Gcn5-TAP and Spt20-MYC and carrying or not a plasmid expressing HA-Ada2. **(E)** Same as in panel (**A**) with wild type or *gcn5Δ* cells expressing Spt20-TAP and Ada2-HA (left panel) or Ada2-TAP and Spt20-HA (right panel).

Supplementary Figure S4. **SAGA subunits sediment with polysomes**. **(A)** Total protein extracts from wild type (WT) or *not5Δ* cells expressing Ada2-TAP, Gcn5-TAP or Spt20-TAP were separated on a 7-47 % sucrose gradient and the polysome profiles detected by an A_{254} nm UV detector are shown in the top panels above the corresponding protein elution analysis. Proteins from the different fractions were TCA precipitated and loaded on SDS-PAGE followed by western blotting with PAP and Rps3 antibodies. **(B)** Total protein extracts from WT cells expressing Ada2-TAP and treated or not with EDTA were separated on 7-47 % sucrose gradient not-treated (-EDTA) or treated (+ EDTA) with EDTA as indicated. Proteins from the different fractions were TCA precipitated and loaded on SDS-PAGE followed by western blotting with PAP and Rps3 antibodies. The numbers of the fractions are indicated above the blots and the positions of free RNAs, 40-60S, 80S and polysomes are indicated under the blots. The densitometric reading is indicated on the top.

Supplementary Figure S5. IP of MS2 from WT polysome fractions. WT cells were transformed with plasmids expressing the mRNAs depicted in (Figure 7A). They were additionally transformed with a plasmid expressing MYC-MS2. Total extracts were loaded on

a sucrose grandient and MS2 was immunoprecipitated from the pooled polysome fractions. RNA was extracted from the immunoprecipitate and the levels of *SPT20*, *GCN5*, *ADA2* and *RPB1* mRNAs were evaluated by RT-qPCR and quantified relative to the amount from the immunoprecipitate of the control.

Supplementary Figure S6. **Tdh3 co-purifies with SAGA subunits. (A)** Gcn5-TAP was purified by single affinity and separated on a 1-20% sucrose gradient. The different fractions were separated by SDS-PAGE and stained by coomassie. The position of Tdh3 and Gcn5-CBP are indicated. **(B)** Total extracts from wild type (WT) or *tdh3Δ* cells or WT cells expressing MYC-tagged Tdh3, and expressing Ada2-TAP were loaded on IgG sepharose. Ada2 was purified by single affinity and the purified proteins were separated by SDS-PAGE followed by coomassie staining. The position of Tdh3, Tdh3-MYC and Ada2-CBP is indicated. **(C)** Total protein extracts from WT, *not5Δ*, *tdh3Δ* and *not5Δ tdh3Δ* expressing Gcn5-MYC were separated by SDS-PAGE (upper panel) or Native-PAGE (lower panel) and the presence of Gcn5-MYC was evaluated by western blotting.

Supplementary Figure S7. Not1 and Tdh3 interact by two-hybrid. Reporter cells expressing Not1 fused to the Gal4 DNA binding domain (pBD-Not1FL) and Tdh3 fused to the Gal4 activation domain (AD) (pAD-Tdh3) or expressed from the same vector with a deletion of the activation domain (pΔAD-Tdh3) or the same vector without any fusion (pAD) were grown exponentially in medium selective for the plasmids and then serially diluted and plated on medium selective for the expression of the reporter plasmid. The only significant growth observed on selective medium was for pBD-Not1-FL with pAD-Tdh3.

Supplementary Figure S8. Blots that were cut to make **Figure 7D** are shown without cutting for a better visualization.

Supplementary Table S1

Strain	Genotype	Source
MY1	*MATα gcn4Δ ura3-52 trp1Δ1 leu2::PET56 gal2*	[1]
MY2332	*MATa leu2Δ20 ura3Δ met15Δ his3Δ1*	BY4741
MY5673	*MATα leu2Δ20 ura3Δ met15Δ his3Δ1 not5::NATMX4 lys2 ARG*	This study
MY6993	*MATa not4::NATMX4 trp1 leu2 ura3 his3*	[2]
MY3939	MY2332; *gcn5::GCN5-13Myc-KANMX4*	This study
MY4856	*MATα leu2Δ20 ura3Δ met15Δ lys2Δ0 his3Δ1 not1::NOT1-TAPTAG-URA3*	Euroscarf
MY4857	*MATα leu2Δ20 ura3Δ met15Δ lys2Δ0 his3Δ1 not4::NOT4-TAPTAG-URA3*	Euroscarf
MY5034	MY1; *spt3::SPT3-TAPTAG-KANMX4*	This study
MY5035	MY1; *spt7::SPT7-TAPTAG-KANMX4*	This study
MY5191	MY1; *taf5::TAF5-TapTag-KANMX4*	This study
MY5320	MY2332; *not5::NOT5-TapTag-KANMX4*	[3]
MY5676	MY4856; *not5::NATMX4*	[3]
MY8728	*MATa ade2 arg4 leu2-3,112 trp1-289 ura3-52 spt20::SPT20-TAPTAG-URA3*	Euroscarf
MY8729	*MATa ade2 arg4 leu2-3,112 trp1-289 ura3-52 spt8::SPT8- TAPTAG -URA3*	Euroscarf
MY8730	*MATa ade2 arg4 leu2-3,112 trp1-289 ura3-52 ada2::ADA2- TAPTAG -URA3*	Euroscarf
MY8731	*MATa ade2 arg4 leu2-3,112 trp1-289 ura3-52 ada1::ADA1- TAPTAG -URA3*	Euroscarf
MY8732	*MATa ade2 arg4 leu2-3,112 trp1-289 ura3-52 ubp8::UBP8- TAPTAG -URA3*	Euroscarf
MY8895	MY5034; *not5::NATMX4*	This study
MY8901	MY5673; *gcn5::GCN5-Myc6-KANMX4*	This study
MY8904	MY5191; *not5::NATMX4*	This study
MY8917	MY5035; *not5::NATMX4*	This study
MY8923	MY8728; *not5::NATMX4*	This study
MY9043	MY8731; *not5::NATMX4*	This study
MY9045	MY8732; *not5::NATMX4*	This study
MY9051	MY8730; *not5::NATMX4*	This study
MY9252	MY8728; *not5::NOT5-HA3-KANMX4*	This study
MY9256	MY8729; *not5::NOT5-HA3-KANMX4*	This study
MY9260	MY8730; *not5::NOT5-HA3-KANMX4*	This study
MY9263	MY8731; *not5::NOT5-HA3-KANMX4*	This study
MY9267	MY8732; *not5::NOT5-HA3-KANMX4*	This study
MY9441	*MATa ada1::KANMX4 his3 leu2 ura3*	Brandl Lab
MY9442	*MATα spt20::KANMX4 his3 leu2 ura3*	Brandl Lab
MY9443	*MATa sgf29::KANMX4 his3 leu2 ura3*	Brandl Lab
MY9444	*MATa sgf11::KANMX4 his3 leu2 ura3*	Brandl Lab
MY9445	*MATa sgf73::KANMX4 his3 leu2 ura3*	Brandl Lab
MY9523	MY8729; *not5::NATMX4*	This study
MY9632	*MATa ade2 arg4 leu2 trp1-289 ura3-52 rpl17b::RPL17B- TAPTAG -URA3*	Euroscarf
MY9646	*MATa ade2 arg4 leu2 trp1-289 ura3-52 taf9::TAF9- TAPTAG -URA3*	Euroscarf
MY9647	*MATa ade2 arg4 leu2 trp1-289 ura3-52 taf10::TAF10- TAPTAG -URA3*	Euroscarf
MY9652	*MATa ade2 arg4 leu2 trp1-289 ura3-52 sgf29::SGF29- TAPTAG -URA3*	Euroscarf
MY9653	*MATa ade2 arg4 leu2 trp1-289 ura3-52 sgf73::SGF73- TAPTAG -URA3*	Euroscarf
MY9704	MY8923; *gcn5::GCN5-MYC13-KANMX4*	This study
MY9747	*MATa gcn5::GCN5-Taptag-HIS3MX4 his3Δ1 leu2Δ0 met15Δ0 ura3Δ0*	Workman Lab
MY9749	*MATa sus1::SUS1- TAPTAG -TRP1*	Workman Lab
MY9751	*MATa ahc1::AHC1-Taptag-HIS3MX4 his3Δ1 leu2Δ0 met15Δ0 ura3Δ0*	Workman Lab
MY9752	*MATa ahc2::AHC2-Taptag-HIS3MX4 his3Δ1 leu2Δ0 met15Δ0 ura3Δ0*	Workman Lab
MY9823	MY9653; *not5::NATMX4*	This study
MY9955	MY9752; *not5::NATMX4*	This study
MY10130	MY9442; *gcn5::GCN5-MYC13-KANMX4*	This study
MY10144	MY9646; *not5::NATMX4*	This study
MY10145	MY9652; *not5::NATMX4*	This study
MY10146	MY9647; *not5::NATMX4*	This study
MY10152	MY9747; *not5::NATMX4*	This study

MY10183	MY9751; not5::NATMX4	This study
MY10383	MY9749; not5::NATMX4	This study
MY10396	MATa his3Δ1 leu2Δ0 met15Δ0 ura3Δ0 tdh3::KANMX4	Euroscarf
MY10397	MATα his3Δ1 leu2Δ0 met15Δ0 ura3Δ0 tdh3::KANMX4	Euroscarf
MY10475	MY10396; ada2::ADA2- TAPTAG -URA3	This study
MY10471	MY10475; not5::NATMX4	This study
MY10661	MY9632; not5::NATMX4	This study
MY11051	MY8728; gcn5::GCN5-Myc13-KANMX4	This study
MY11315	MY9704; tdh3::KANMX4	This study
MY11316	MY10396; gcn5::GCN5- TAPTAG -HIS3MX4	This study
MY11319	MY11316; not5::NATMX4	This study
MY11320	MY9747; spt20::SPT20-Myc13-KANMX4	This study
MY11323	MY11320; not5::NATMX4	This study
MY11328	MY9704; ada2::ADA2-HA3-TRP1	This study
MY11355	MY11051; tdh3::KANMX4	This study
MY11409	MY11051; ada2::ADA2-HA3-HISMX4	This study
MY11426	MY9747; ada2::ADA2-Myc13-KANMX4	This study
MY11427	MY11426; not5::NATMX4	This study
MY11452	MY9747; spt20::KANMX4	This study
MY11467	MY11320; ada2::TRP1	This study
MY11470	MY9442; ada2::ADA2- TAPTAG-URA3	This study
MY11510	MY8728; ada2::TRP1	This study
MY11512	MY8728; gcn5::TRP1	This study
MY11513	MY8730; gcn5::TRP1	This study
MY11763	MATa trp1-901 leu2-3,112 ura3-52 his3-200 gal4Δ gal80Δ LYS2:: GAL1-HIS3 GAL2-ADE2 met2:GAL7-lacZ sir3::NATMX4 sir4::URA3 sir2::HYGMX4	(YSH625 [4])
MY11767	MY11763; pBD-TRP1 + pAD-TDH3-ΔAD-LEU2	[4]
MY11768	MY11763; pBD-TRP1 + pAD-TDH3-AD-LEU2	[4]
MY11862	MY11763; pBD-Not1-TRP1 + pAD-LEU2	This study
MY11864	MY11763; pBD-Not1Cterm-TRP1 + pAD-LEU2	This study
MY11867	MY11763; pBD-Not1-TRP1 + pAD-TDH3-ΔAD-LEU2	This study
MY11869	MY11763; pBD-Not1Cterm-TRP1 + pAD2-TDH3-ΔAD-LEU2	This study
MY11870	MY11763; pBD-Not1-TRP1 + pAD-TDH3-LEU2	This study
MY11872	MY11763; pBD-Not1Cterm-TRP1 + pAD-TDH3-LEU2	This study
MY11922	MY9747; not4::NATMX4	This study
MY11926	MY8728; not4::NATMX4	This study
MY11928	MY8730; not4::NATMX4	This study
MY11948	MY2332; not4::NOT4 (1-1290)- TAPTAG- KanMX4	This study
MY11975	MY2332; tdh3::TDH3- TAPTAG- KanMX4	This study
MY12008	MY11975; not4::NATMX4	This study
MY12062	MY11975; not5::NATMX4	This study
MY12191	MY4857; ada2::KanMX4	This study
MY12121	MY11512; ada2::ADA2-HA3-KANMX4	This study
MY12123	MY11513; spt20::SPT20-HA3-KANMX4	This study
MY12148	MY8728; ada2::ADA2-HA3-KANMX4	This study
MY12157	MY8730; spt20::SPT20-HA3-TRPMX4	This study
MY12150	MY8730; gcn5::KANMX	This study
MY12213	MY2332; pMAC1020 + pMAC1105	This study
MY12215	MY2332; pMAC1078 + pMAC1105	This study
MY12217	MY5673; pMAC1020 + pMAC1105	This study
MY12219	MY5673; pMAC1078 + pMAC1105	This study
MY12278	MY12157; Sgf73::KANMX4	This study

Supplementary Table S2

Name	Sequence
ADA2.F2	GAATAGAATATAGCATTTTTTCCAGAGCCAGAATTGGATGCGGATCCCCGGGTTAATTAA
ADA2.R1	TAACTAGTGACAATTGTAGTTACTTTTCAATTTTTTTTTTGGAATTCGAGCTCGTTTAAAC
ADA2.F1	TATCAGCGTAGTCTGAAAATATATACATTAAGCAAAAAGACGGATCCCCGGGTTAATTAA
ADA2.RT1	TGCACATAGGTCGAACGCAG
ADA2.RT2	GTTGCTGCTCGTCGTTGGAC
ADA2.REC1	GAATTCGATATCAAGCTTATCGATACCGTCGACAATGTCAAACAAGTTTCACTGTGACGT
ADA2.REC2	GCGTGACATAACTAATTACATGACTCGAGGTCGACTTACATCCAATTCTGGCTCTGGA
FUR4.REC5	AACAAAAGCTGGAGCTCGTTTAAACGGCGCCGCGCCTAGACAAGCGCGAGGAGGAA
FUR4.REC6	CGGGACGTCATACATACTAGTGCGGCCGCTATTCCCTCCTATTCTTATTATGC
GCN5.F2	TAAAGTAAAAGAAATACCTGAATATTCTCACCTTATTGATCGGATCCCCGGGTTAATTAA
GCN5.R1	TCTTCGAAAGGAATAGTAGCGGAAAAGCTTCTTCTACGCAGAATTCGAGCTCGTTTAAAC
GCN5.F1	CCAAAAGTCTTCAGTTAACTCAGGTTCGTATTCTACATTAGCGGATCCCCGGGTTAATTAA
GCN5.V4	GGCTCTGAAGGAGCACAAG
GCN5.RT1	GGTGCTCAAGTCCATTGGCT
GCN5.RT2	CGAGATGGATGCGTTGGCAC
GCN5.REC1	GAATTCGATATCAAGCTTATCGATACCGTCGACAATGGTCACAAAACATCAGATTGAAGAGGATC
GCN5.S1	TAAAGTAAAAGAAATACCTGAATATTCTCACCTTATTGATTCTCACGAAAAGAGAAGATGGAAG
GCN5.S2	CTTCGAAAGGAATAGTAGCGGAAAAGCTTCTTCTACGCAATCGATGAATTCGAGCTCG
SGF11.S1	GCAGCTCATTTACAGAGATGTTTGAGTAGGGGTGCTAGACGTCTTCACGAAAAGAGAAGATGGAAG
SGF11.S2	CTGTCTGTGCCTTTTCAATTACCCATAAACCACCACCTAGTGATCGATGAATTCGAGCTCG
SPT20.F1	AAGGAATAGTTACGGTTAATTTGCGCCTATATATTTCAGGGCGGATCCCCCGGTTAATTAA
SPT20.R1	TATATATATATATAAGGAATGATAACTCTATTTAAGTAGAGAATTCGAGCTCGTTTAAAC
SPT20.F2	AACAGAGCGCAAGTAGCACGCCCTCTTCTACTACAATGTCACGGATCCCCGGTTAATTAA
SPT20.RT1	GGCCTTGGAGCAACAACAG
SPT20.RT2	CTGGTGTACCGCTCTCGCTT
SPT20.REC1	GAATTCGATATCAAGCTTATCGATACCGTCGACAATGAGTGCCAATAGCCCGACAGGAAAC
SPT20.REC2	GCGTGACATAACTAATTACATGACTCGAGGTCGACTTATGACATTGTAGTAGAAGAGGGCGTG
TDH3.S1	ACCAGAGTTGTCGACTTGGTTGAACACGTTGCCAAGGCTTCTCACGAAAAGAGAAGATGGAAG
TDH3.S2	GAAAATTTATTTAAATGCAAGATTTAAAGTAAATTCACATCGATGAATTCGAGCTCG
MS2BS.CYC1 FOR	ACACCGATTATTTAAAGCTGCAGGTCGACCTCGAGCTCGGATCCACTAGTAACGGCC
MS2.REC1	GAATTCGATATCAAGCTTATCGATACCGTCGACA ATGGACTACAAAGACGATGACGACAAG
MS2.REC2	GCGTGACATAACTAATTACATGACTCGAGGTCGACGATATCGTAGATGCCGGAGTTTGC
Not4. 5‘	ACTGAATCCTAACCTCCCAAA
Not4. V4	TGGATAGCGAACAACAACACG
NOT4-1290.S1	AGATCCTTATGACGCACTAGGGAATGCTGTTGACTTTTTGTCTCACGAAAAGAGAAGATGGAAG
NOT4-1290.S2	GGCGCTTCTGATAATTTGATAGAGAATGTAGTCTTGCATCGATGAATTCGAGCTCG
NOT5.V1	ACAACGTAGATCGCTGCTGG
NOT5.V4	GGGGAAAGCCCTGATCATCG
NOT5.NAT.R1	GTAAATCACGATGAGAATTATATAAGTAAAAGGAAACTGTGCATAGGCCACTAGTGGATCTG
NOT5.NAT.R1	TATTTTTTATTGATTGCATGAAACATCCGCTCATTCTGTCCAGCTGAAGCTTCGTACGC
RPB1.RT1	GTCACCAAGTTACAGCCCAACG
RPB1.RT2	AGATCCTGGGCTGTAGCCTG
NIP1.RT1	AGCTGATGAGCGTGCTAGAC
NIP1.RT2	AGGAACGACGAATGGATTTTGGAG

Supplementary Table S3		
**Plasmid name**	**Description**	**Parent plasmid**
pMAC724	p*SPT3*p-7HA-*NOT2*-*CYC1*t-*LEU2*	pGREG535 [5]
pMAC699	p*RPS7A*p-6MYC-*NOT5 (1-560)*-*CYC1*t-*URA3*	pGREG561 [5]
pMAC1020	*ADA2* ORF amplified via REC1 and REC2 to GAP repair linearized pMAC724 via *Sal*I	pMAC724
pMAC1024	*SPT20* ORF amplified via REC1 and REC2 to GAP repair linearized pMAC724 via *Sal*I	pMAC724
pMAC1078	MS2bs amplified via MS2BS.CYC1 FOR and MS2BS.CYC1.REV from pCDNA3-MS2bs [6] to GAP repair linearized pMAC1020 via *Xho*I	pMAC1020
pMAC1081	MS2 amplified via REC1 and REC2 from pFLAG-MS2-hmGFP [6] to GAP repair linearized pMAC699 via *Sal*I	pMAC699
pMAC1105	*FUR4* PROMOTER amplified via REC5 and REC6 to GAP repair linearized pMAC1081 via (*Asc*I-*Not*I)	pMAC1081

1 Collart, M. A. & Struhl, K. NOT1(CDC39), NOT2(CDC36), NOT3, and NOT4 encode a global-negative regulator of transcription that differentially affects TATA-element utilization. *Genes Dev* **8**, 525-537 (1994).

2 Panasenko, O. O. & Collart, M. A. Not4 E3 ligase contributes to proteasome assembly and functional integrity in part through Ecm29. *Mol Cell Biol* **31**, 1610-1623, doi:10.1128/MCB.01210-10 (2011).

3 Villanyi, Z. *et al.* The Not5 subunit of the ccr4-not complex connects transcription and translation. *PLoS Genet* **10**, e1004569, doi:10.1371/journal.pgen.1004569 (2014).

4 Ringel, A. E. *et al.* Yeast Tdh3 (glyceraldehyde 3-phosphate dehydrogenase) is a Sir2-interacting factor that regulates transcriptional silencing and rDNA recombination. *PLoS Genet* **9**, e1003871, doi:10.1371/journal.pgen.1003871 (2013).

5 Jansen, G., Wu, C., Schade, B., Thomas, D. Y. & Whiteway, M. Drag&Drop cloning in yeast. *Gene* **344**, 43-51, doi:10.1016/j.gene.2004.10.016 (2005).

6 Gong, C. & Maquat, L. E. lncRNAs transactivate STAU1-mediated mRNA decay by duplexing with 3' UTRs via Alu elements. *Nature* **470**, 284-288, doi:10.1038/nature09701 (2011).

Supplementary Table S4. Proteins purifying with Gcn5 were identified by mass spectrometry

		Spectral counts				Protein probabilities				Percentage of spectra				Unique peptide counts				Percentage coverage			
		WT		*not5 Δ*		WT		*not5 Δ*		WT		*not5 Δ*		WT		*not5 Δ*		WT		*not5 Δ*	
Name	MW	TP	SAGA	TP	SAGA	TP	SAGA	TP	SAGA	TP	SAGA	TP	SAGA	TP	SAGA	TP	SAGA	TP	SAGA	TP	SAGA
Tra1	433 kDa	59	17	42	7	100%	100%	100%	100%	1.60%	1.90%	1.60%	1.10%	52	11	41	6	17%	3.30%	12%	1.80%
Spt7	153 kDa	38	15	31	7	100%	100%	100%	100%	1.10%	1.70%	1.20%	1.10%	31	9	28	6	24%	9.50%	19%	5%
Ada3	79 kDa	22	26	24	10	100%	100%	100%	100%	0.61%	3.00%	0.91%	1.60%	20	12	18	7	35%	22%	29%	11%
Taf12	61 kDa	14	25	9	24	100%	100%	100%	100%	0.39%	2.80%	0.34%	3.80%	10	10	8	11	22%	24%	20%	28%
Taf5	89 kDa	20	17	18	12	100%	100%	100%	100%	0.55%	1.90%	0.68%	1.90%	16	10	14	8	28%	16%	21%	12%
Spt20	68 kDa	12	14	8	13	100%	100%	100%	100%	0.33%	1.60%	0.30%	2.10%	12	7	6	8	24%	16%	12%	19%
Sgf73	73 kDa	11	11	11	9	100%	100%	100%	100%	0.30%	1.30%	0.42%	1.40%	11	7	10	6	19%	15%	18%	13%
Taf6	58 kDa	13	0	10	3	100%	0	100%	100%	0.36%	0	0.38%	0.48%	11	0	10	2	26%	0	22%	3.70%
Gcn5	51 kDa	13	0	12	3	100%	0	100%	100%	0.36%	0	0.45%	0.48%	10	0	9	2	26%	0	25%	4.80%
Spt8	66 kDa	11	3	9	0	100%	100%	100%	0	0.30%	0.34%	0.34%	0	10	2	9	0	18%	3.50%	18%	0
Ada2	51 kDa	12	0	7	0	100%	0	100%	0	0.33%	0	0.26%	0	9	0	6	0	27%	0	19%	0
Spt3	39 kDa	7	5	6	0	100%	100%	100%	0	0.19%	0.57%	0.23%	0	7	2	4	0	24%	9.80%	17%	0
Sgf29	29 kDa	6	2	5	4	100%	100%	100%	100%	0.17%	0.23%	0.19%	0.64%	5	2	4	3	27%	11%	22%	15%
Ada1	54 kDa	7	0	11	0	100%	0	100%	0	0.19%	0	0.42%	0	6	0	8	0	11%	0	17%	0
Ubp8	54 kDa	10	0	4	0	100%	0	100%	0	0.28%	0	0.15%	0	10	0	4	0	41%	0	36%	0
Taf9	17 kDa	4	0	3	0	100%	0	100%	0	0.11%	0	0.11%	0	4	0	3	0	22%	0	7.60%	0
Taf10	23 kDa	2	0	0	0	100%	0	0	0	0.06%	0	0	0	2	0	0	0	21%	0	0	0

Gcn5 was purified by affinity purification. The total purification (TP) was separated in 2. Half was analyzed by mass spectrometry and half was loaded on a sucrose gradient.

Fractions 6 and 7 of the sucrose gradient (containing SAGA) were analyzed by mass spectrometry.

The results are indicated with a protein threshold of 95% and a minimum of 2 peptides. The total purification of Gcn5 (TP) was analyzed once by mass spectrometry, and the SAGA fractions twice.

Sus1 and Sgf11 were not detected.

The complete data sets for this threshhold are indicated as Supplementary file S5.

Gcn5 is more present in SAGA fractions from *not5 Δ* than from the wild type, but most SAGA subunits instead are less present

Globally Gcn5 is purified relatively similarly from *not5 Δ* as from the wild type but represents a higher percentage of total spectra in the purification from the mutant

Supplementary Table S5. Scaffold file with the mass spectrometry results of the purification of Gcn5-TT from wild type cells (Sample 1, LM_1) and SAGA fractions from the sucrose gradient factionation of this purification (Sample 1, LM_1bis) and the purification of Gcn5-TT from *not5Δ* cells (Sample 2, LM_2) and SAGA fractions from the sucrose gradient factionation of this purification (Sample 2, LM_2bis). Scaffold file is available at the publisher's website.
