# Supplementary figures and images for: Not5-dependent co-translational assembly of Ada2 and Spt20 is essential for functional integrity of SAGA
Source: Nucleic Acids Res. 2016 Nov 29;45(3):1186–99. doi: 10.1093/nar/gkw1059 (PMC5388395; doi:10.1093/nar/gkw1059)

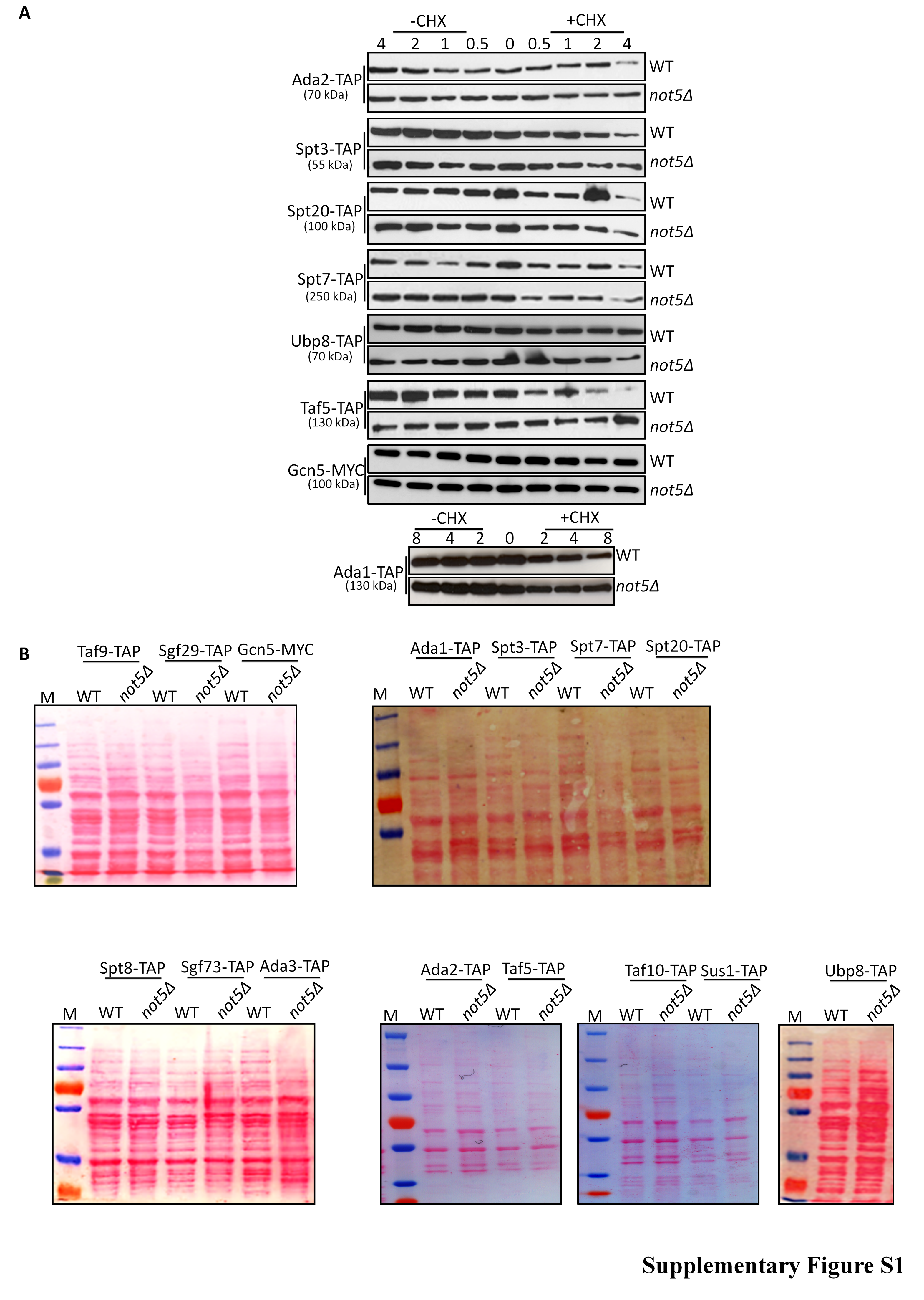

Supplement: Supplementary Data [file gkw1059_Supp.zip › nar-01067-v-2016-File012.tif]

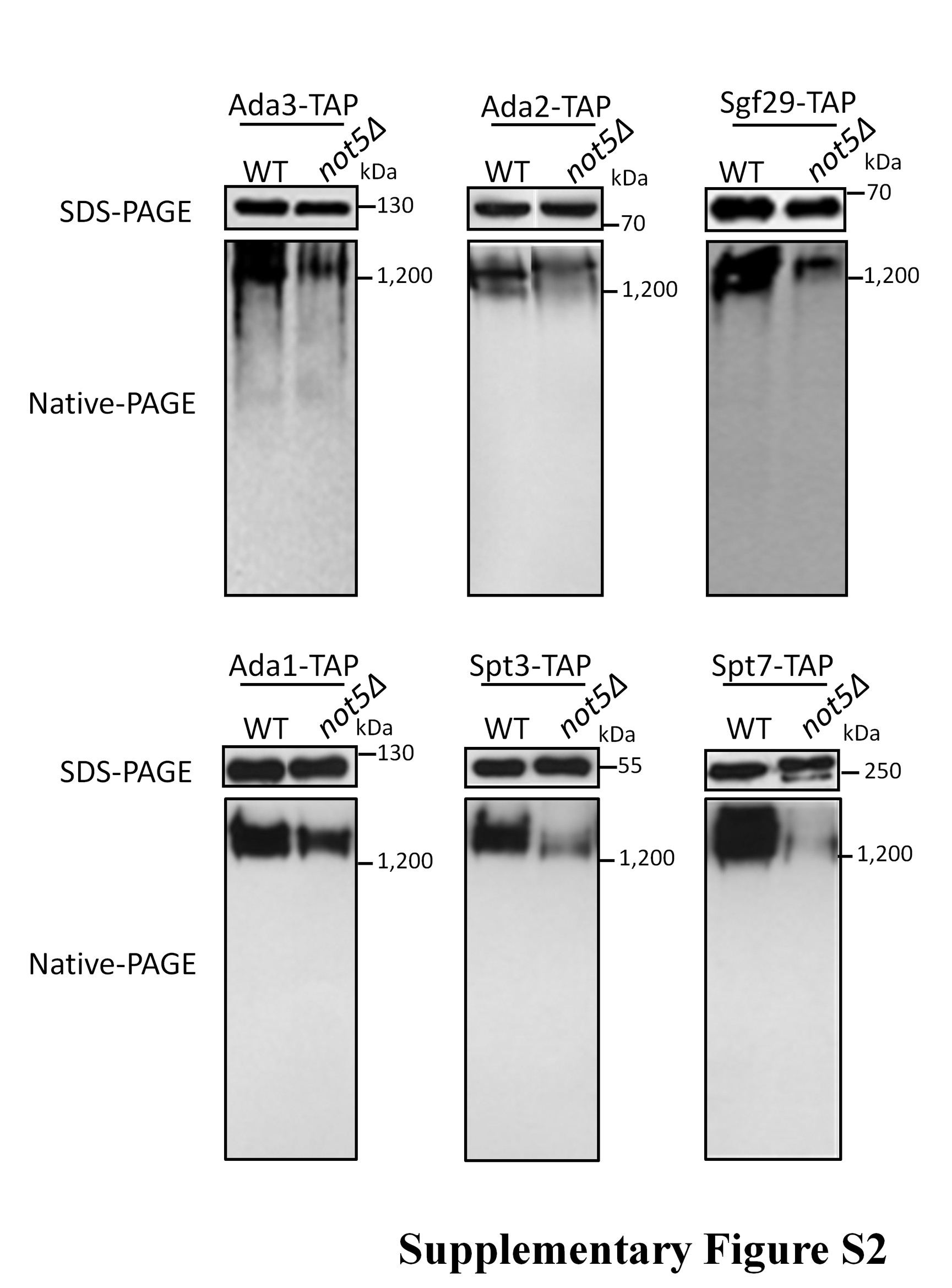

Supplement: Supplementary Data [file gkw1059_Supp.zip › nar-01067-v-2016-File013.tif]

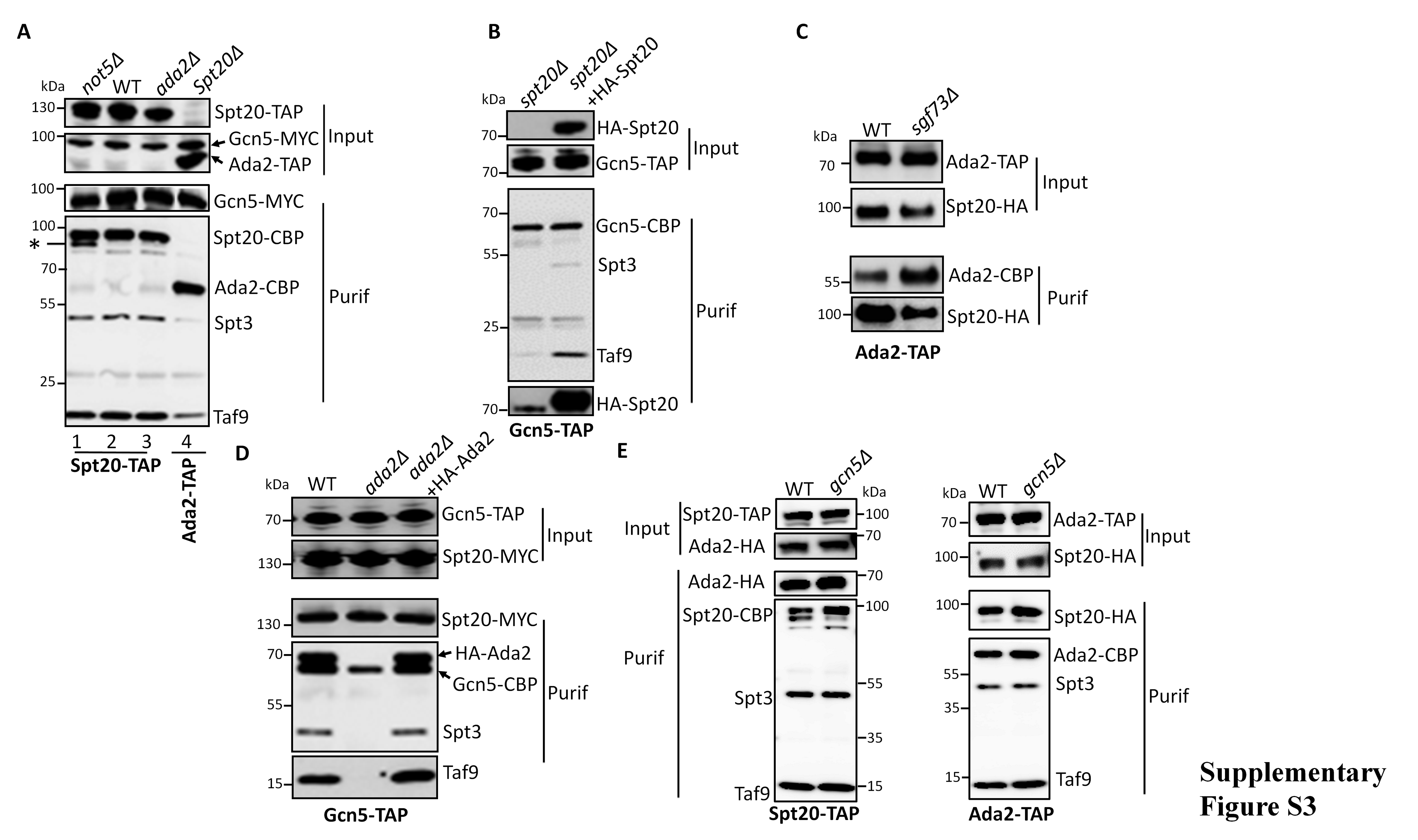

Supplement: Supplementary Data [file gkw1059_Supp.zip › nar-01067-v-2016-File014.tif]

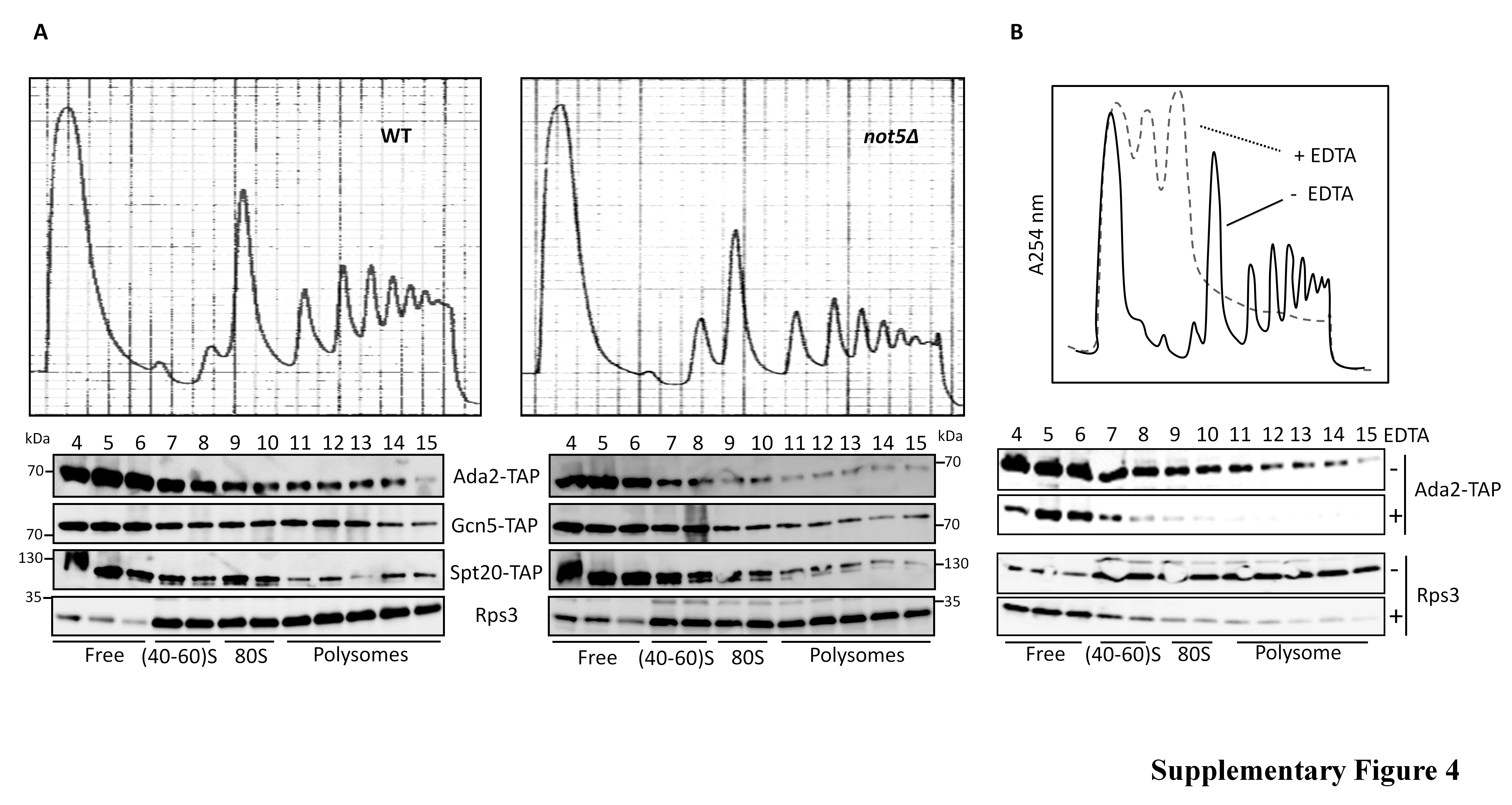

Supplement: Supplementary Data [file gkw1059_Supp.zip › nar-01067-v-2016-File015.tif]

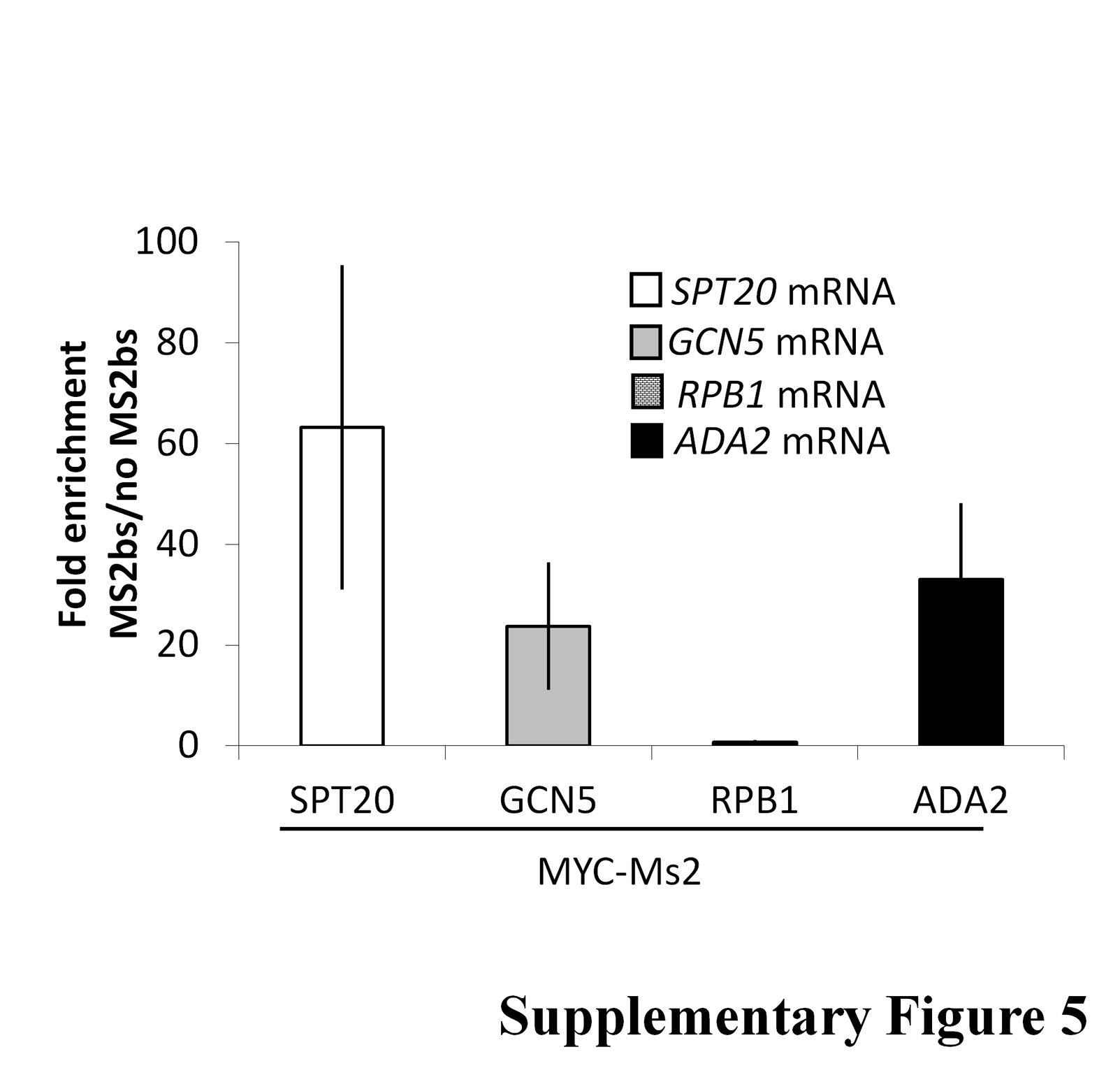

Supplement: Supplementary Data [file gkw1059_Supp.zip › nar-01067-v-2016-File016.tif]

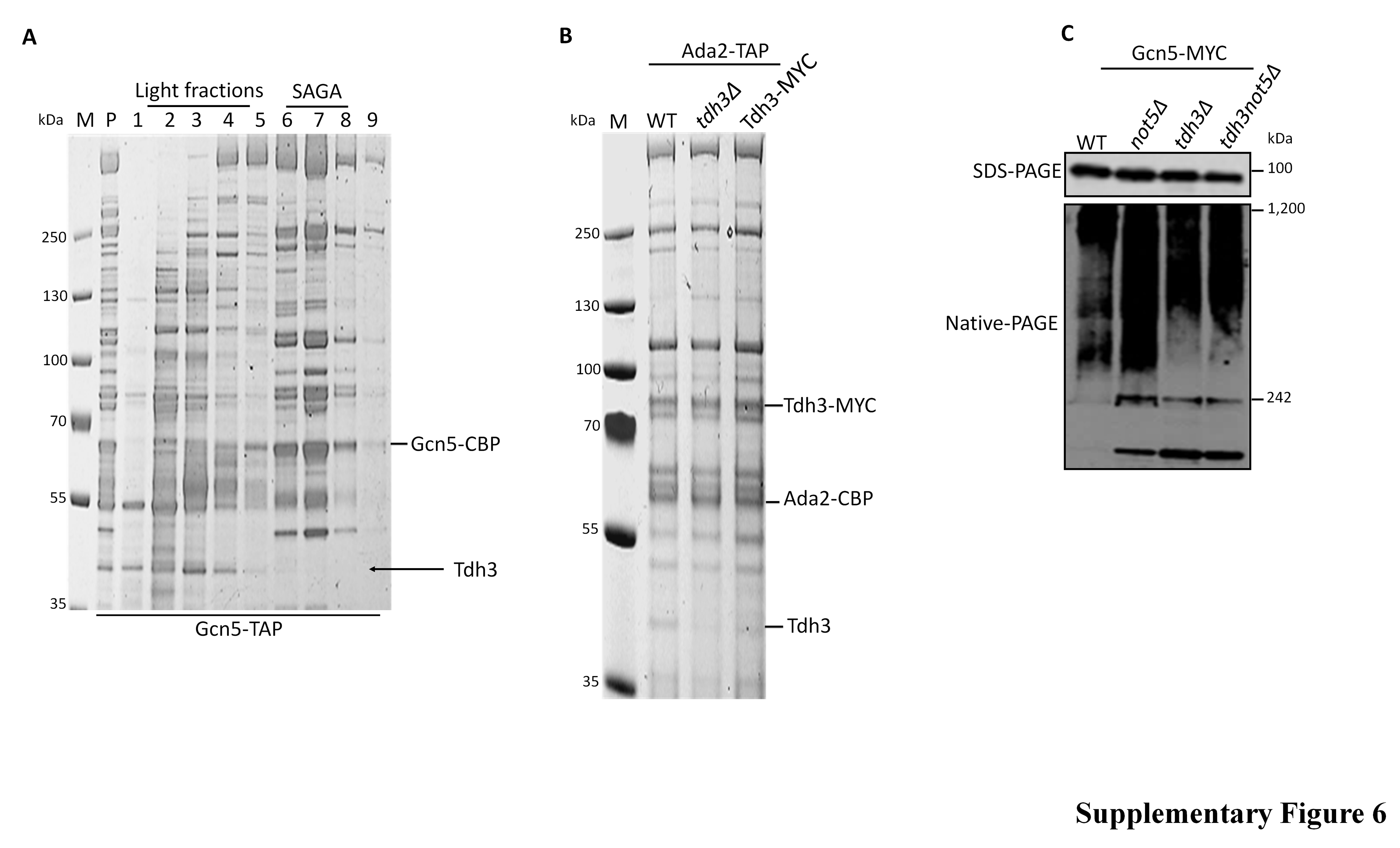

Supplement: Supplementary Data [file gkw1059_Supp.zip › nar-01067-v-2016-File017.tif]

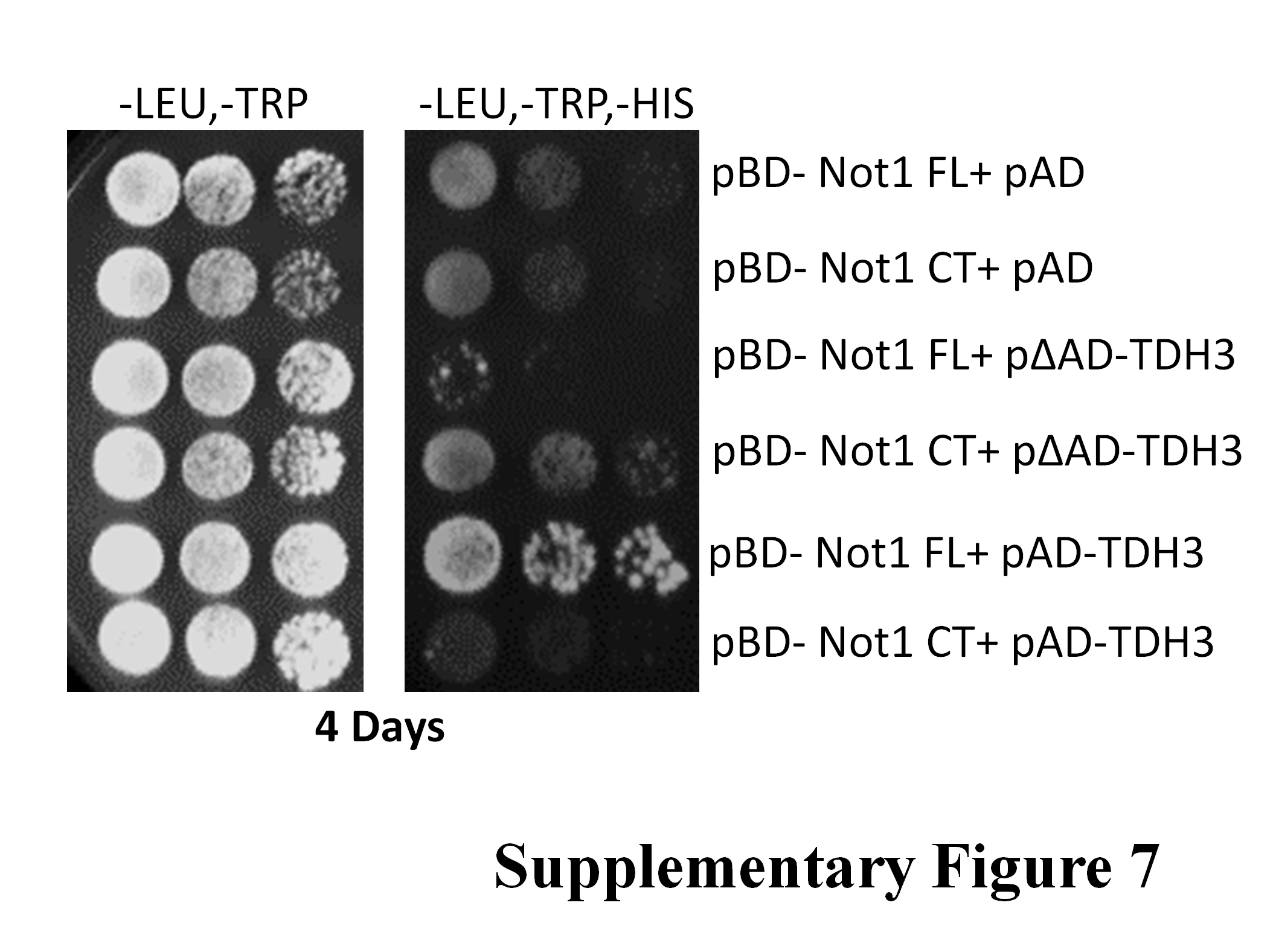

Supplement: Supplementary Data [file gkw1059_Supp.zip › nar-01067-v-2016-File018.tif]

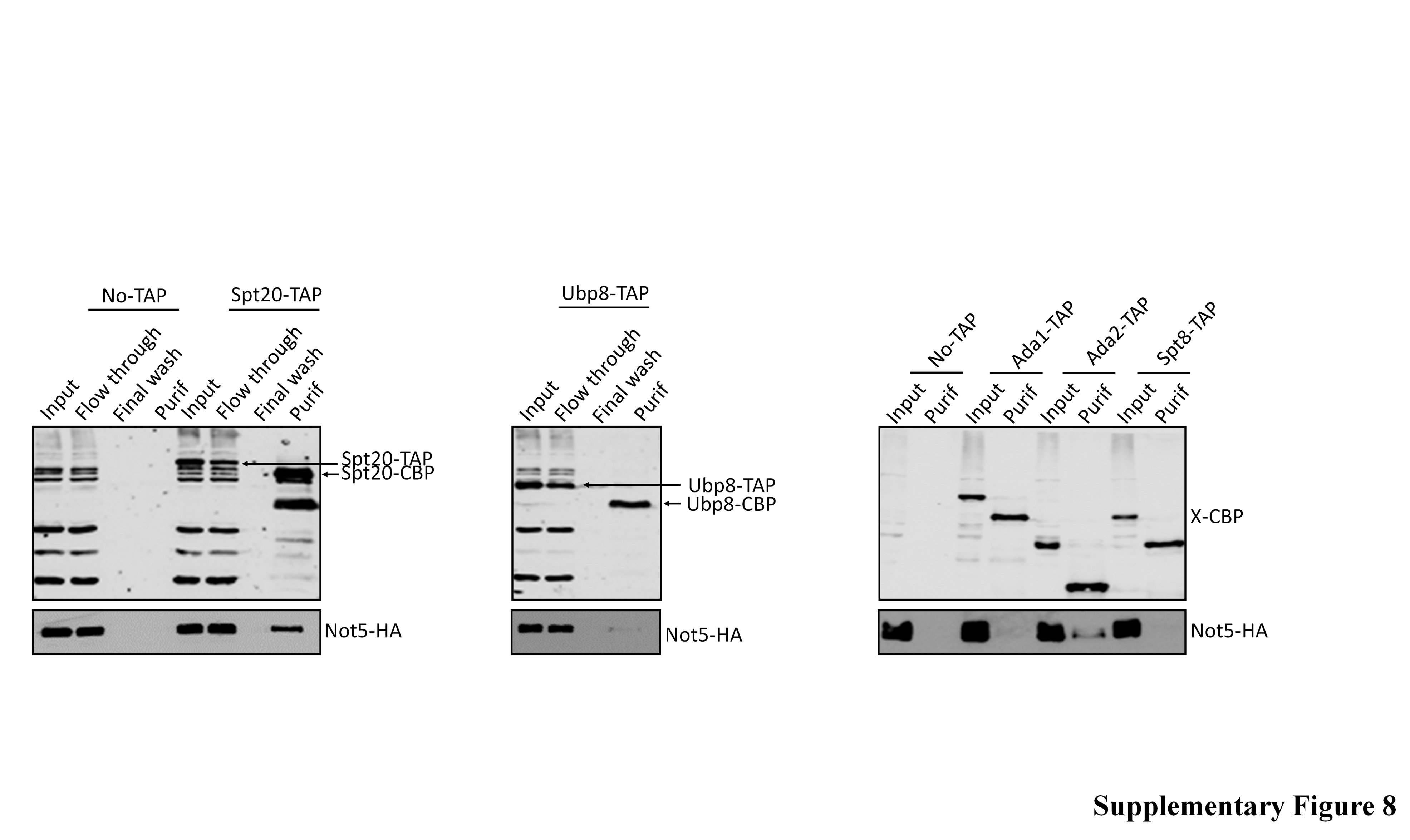

Supplement: Supplementary Data [file gkw1059_Supp.zip › nar-01067-v-2016-File019.tif]
